# Replication and transcription machinery for ranaviruses: components, correlation, and functional architecture

**DOI:** 10.1186/s13578-021-00742-x

**Published:** 2022-01-06

**Authors:** Fei Ke, Xue-Dong Yu, Zi-Hao Wang, Jian-Fang Gui, Qi-Ya Zhang

**Affiliations:** 1 State Key Laboratory of Freshwater Ecology and Biotechnology, Institute of Hydrobiology, College of Modern Agriculture Sciences, University of Chinese Academy of Sciences, Chinese Academy of Sciences, Wuhan, 430072 China; 2 The Innovation Academy of Seed Design, Chinese Academy of Sciences, Beijing, 100101 China

**Keywords:** Ranavirus, Nucleocytoplasmic large DNA viruses (NCLDVs), Viral replication and transcription machinery, DNA polymerase, Proliferating cell nuclear antigen (PCNA), Single-stranded DNA binding protein, RNA polymerase

## Abstract

**Background:**

Ranaviruses (family *Iridoviridae*) are promiscuous pathogens that can infect across species barriers in poikilotherms and can replicate in amphibian and fish cells and even in cultured mammalian cells. However, as nucleocytoplasmic large DNA viruses (NCLDVs), their replication and transcription mechanisms remain largely unknown. Here, we screened and uncovered the replication and transcription machinery of two ranaviruses, *Andrias davidianus* ranavirus (ADRV) and *Rana grylio* virus (RGV), by a combination of methods, including the isolation of proteins on nascent DNA, recombinant virus-based affinity, and NanoLuc complementation assay.

**Results:**

The ranavirus replication and transcription machinery was deeply dissected and identified as a complicated apparatus containing at least 30 viral and 6 host proteins. The viral proteins ADRV-47L/RGV-63R (DNA polymerase, vDPOL), ADRV-23L/RGV-91R (proliferating cell nuclear antigen, vPCNA), ADRV-85L/RGV-27R (single-stranded DNA binding protein, vSSB), ADRV-88L/RGV-24R (vhelicase/primase), etc., constitute the core replisome. Specifically, the core of the transcription complex, the viral RNA polymerase, contain the host RNAPII subunits Rpb3, Rpb6, and Rpb11, which was a first report in NCLDVs. Furthermore, correlations and interactions among these factors in the machinery were described. Significantly, the replisome core protein vDPOL (ADRV-47L) can interact with numerous viral and host proteins and could act as a linker and regulation center in viral DNA replication and transcription. Thus, these results depicted an architecture for ranavirus replication and transcription.

**Conclusions:**

Up to 36 components from ranavirus and their host were found to form viral replisomes and transcription complexes using a series of precise methods, which further constructed an architecture for ranavirus replication and transcription in which vDPOL was a key central factor and various components correlated and cooperated. Therefore, it provides a cornerstone for further understanding the mechanisms of the replication and transcription of ranaviruses which can ensure the efficient production of progeny virus and adaptation to cross-species infection.

**Supplementary Information:**

The online version contains supplementary material available at 10.1186/s13578-021-00742-x.

## Background

Members of the genus *Ranavirus* (family *Iridoviridae*) are nucleo-cytoplasmic large DNA viruses (NCLDVs) and have been considered as promiscuous pathogens [1], after isolation from aquatic animals including reptiles [2, 3], amphibians [4–6], and bony fish [7–10]. These viruses represent a great threat to economically and ecologically important poikilotherms [11]. Even more important is that many ranaviruses can infect more than one host species and cross species barriers [12–16]. The ranaviruses that have been sequenced have a genome with a unit size of 104–140 kbp, which encodes approximately 100–160 potential open reading frames (ORFs) [7].

Genome replication and transcription are central processes for all living organisms [17]. DNA replication is carried out by a complex called the “replisome” containing numerous proteins, among which the core proteins include a helicase, primase, DNA polymerase (DPOL), sliding clamp (processivity factor), clamp loader, and single-stranded binding (SSB) protein [18, 19]. These core replisome proteins function in a highly choreographed fashion to propagate the replication fork. In addition to the core proteins, several other proteins act before or after the fork to change the DNA conformation, repair and ligate Okazaki fragments, and so on. DNA transcription to mRNA is usually carried out by DNA-dependent RNA polymerase II (RNAP II) in eukaryotes. RNAP II contains 12 or more subunits with a core enzyme consisting of 5 subunits: two large subunits and three smaller subunits [20, 21]. As DNA viruses, they have genome replication and transcription processes that follow the general rules of their hosts but with some differences. For example, because of the limitation of genome size, viruses usually do not encode all the core enzymes needed for replication or transcription but hijack host proteins, or a viral protein can function as two enzymes. In addition, DNA virus genome replication is usually coupled with transcription [22].

Unlike other extensively researched NCLDVs in mammals, the components required for replication and transcription in ranaviruses have been mainly predicted by bioinformatic analysis of viral genomes. The predicted proteins include DPOL, replication factor, NTPase/helicase-like protein, RAD2 DNA repair protein, RNA polymerase subunits, and proteins involved in nucleic acid metabolism [5]. Although some of these proteins have been investigated [23–29], the detailed mechanisms of genome replication and transcription of ranaviruses remain largely an enigma. For example, the number of these potential proteins is still less than the number needed to complete the DNA replication and transcription cycles, and the predicted ones need to be proven experimentally. The viral and host proteins that participate in specific processes, such as resolving DNA topology, and how they are organized are still unknown.

In recent years, a method known as iPOND (isolation of proteins on nascent DNA) was developed and used for isolating and identifying proteins on replicating DNA [30]. The method relies on labeling replicating DNA with deoxynucleoside analogs, such as 5-ethynyl-2′-deoxyuridine (EdU). A fluorescent-azide or biotin-azide conjugate can then be linked to the ethynyl group through the click chemistry reaction, enabling the labeled DNA to be visualized by microscopy or affinity purified with streptavidin beads. The replicating DNA-associated proteins can be cross-linked to the DNA with formaldehyde, copurified with the labeled DNA, and identified by mass spectrometry (MS). This method has been used to identify the replisome and transcriptome proteins of mammalian viruses, including herpes simplex virus type 1 (HSV-1) [31, 32], adenovirus type 5 (Ad5) [33], vaccinia virus (VACV) [34], and Epstein–Barr virus (EBV) [35]. Nevertheless, the application of this method in cells from lower vertebrates or aquatic animals has not been reported.

Previously, we characterized two ranaviruses: *Rana grylio* virus (RGV) from diseased pig frog *Rana grylio* and *Andrias davidianus* ranavirus (ADRV) from diseased Chinese giant salamander *Andrias davidianus* [4, 6]. RGV has high similarity with frog virus 3 (FV3) in genome sequence and architecture. The latter is the type species of *Ranavirus* [1]. ADRV has a different genome architecture than RGV but high sequence similarity in viral proteins [6]. In the present study, we employed iPOND and recombinant virus-based affinity purification coupled with MS analysis to identify replication- and transcription-related proteins in the two ranaviruses. The use of these methods in the amphibian cell culture system was successful, and numerous viral and host factors, including those not reported previously, were obtained. In the following functional analysis, important factors and core replisome proteins in ranavirus replication, including the SSB protein and topoisomerase, were first verified, as well as virus transcription complex-related proteins.

## Results

### EdU labeling conditions and iPOND application feasibility

**Fig. 1:**
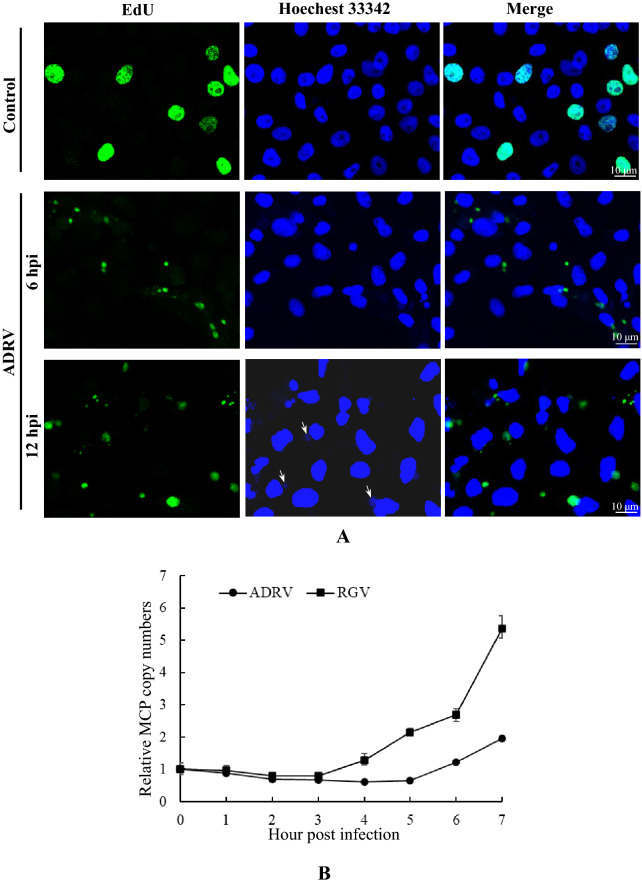
Determination of the conditions for iPOND in ADRV- and RGV-infected GSTC cells. **A** Visualization of DNA labeled by EdU in ADRV infections at 6 and 12 hpi. GSTC cells were infected with or without ADRV at 1 MOI. The cells were incubated for 1 h with 10 μM EdU at the indicated time points and then fixed, permeabilized, and reacted with Alexa Fluor 488 azide. Cellular DNA was stained with Hoechst 33342 (blue). The EdU-labeled DNA presented is shown in green color. Visible Hoechst-labeled cytoplasmic viral factories are indicated with arrows. **B** Kinetics of DNA synthesis of the two viruses. GSTC cells were exposed to ADRV and RGV for 1 h at 0.1 MOI, respectively, and collected at the indicated time points. Total DNA was extracted. Viral genome amounts were determined by qPCR detection of MCP copy numbers. The genome amounts after 1 h of adsorption were set as 1

EdU labeling and its conditions were examined in ADRV and RGV infected Chinese giant salamander thymus cells (GSTC) to confirm the feasibility of iPOND application in lower vertebrate cells. In cells without infection, EdU fluorescence was detected in the nuclei of approximately 25% of cells, indicating cellular genome replication within these cells. At 6 h post infection (hpi), it was not detected in almost all cell nuclei, and only a very weak signal appeared in a small number of nuclei. In contrast, green fluorescence appeared mainly in the cytoplasm in an aggregated form. At 12 hpi, more EdU signals were detected in the cytoplasm, and no signal was detected in the nuclei. The size and fluorescence intensity of the EdU-labeled cytoplasmic punctate area increased from 6 to 12 hpi. The signals from Hoechst 33342-stained viral factories were also merged with the EdU labeling. Increasing EdU signals were synchronized with the formation of viral factories which has been identified in our previous study [26, 36]. Similar observations were obtained from the two virus (ADRV and RGV) infections (Fig. 1A, Additional file 1: Fig. S1). The results showed that EdU can be used for labeling viral nascent DNA in cells from poikilotherm.

EdU labeling during 1 h intervals was also performed under ADRV infection to observe the progress of labeling. The results showed that a small number of cytoplasmic foci appeared at 3 to 4 hpi, which were fewer than the strongly labeled nuclei. As time increased, the number of cytoplasmic foci increased, as did the size and fluorescence intensity. No nucleus was labeled by EdU at 6–7 hpi (Additional file 1: Fig. S2).

Then, the EdU labeling time and the added time point were determined. At the three tested labeling times (10, 20, and 30 min), the detected EdU signals grew stronger as the labeling time increased. In the samples labeled for 30 min, there were abundant cytoplasmic fluorescence signals with detectable intensity (Additional file 1: Fig. S3). Therefore, a 30 min labeling time was used in the following experiments. To select a time point for EdU labeling in iPOND, the genome replication kinetics of the two viruses were determined by qPCR. The contents of viral DNA decreased in the first 2 h after 1 h of adsorption for both viruses (Fig. 1B). For ADRV, its DNA content increased slightly from 4 to 5 hpi and increased clearly after 5 hpi, while the DNA content of RGV increased clearly after 3 hpi. Although the replication of the RGV genome started earlier than that of ADRV, the time point 7 hpi was selected as the labeling time point for both viruses based on the EdU labeling observation and genome replication kinetics described above, and considering operational convenience.

### Screening of viral proteins associated with nascent DNA

**Fig. 2:**
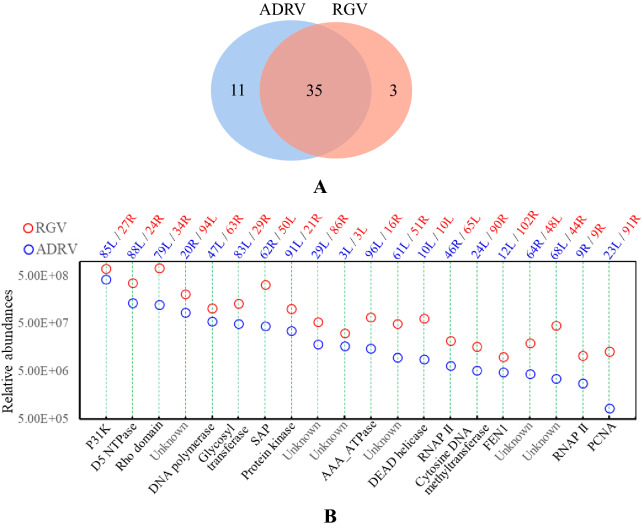
Viral proteins associated with nascent DNA from the iPOND-MS analysis. **A** Numbers of viral proteins obtained from ADRV- and RGV-infected samples. There are 46 ADRV proteins and 38 RGV proteins. Thirty-five pairs of proteins were shared by the two viruses. **B** Relative abundances of the 20 pairs of proteins detected in all assays among the 35 pairs of proteins. The proteins encoded by ADRV are marked in blue, and those encoded by RGV are marked in red. Their possible functions or contained domains are labeled at the bottom

Three independent iPOND-MS experiments containing click and nonclick controls were performed for each virus in GSTC cells. A total of 46 ADRV proteins and 38 RGV proteins were screened in parallel experiments (Table 1). Among them, 35 ADRV proteins were also found in RGV samples (Fig. 2A). Based on previous reports or the contained domains, these proteins can be divided into several categories, including those involved in nucleotide precursor metabolism, DNA replication, genome modification, transcription, and DNA or RNA processing. There were also several proteins that had a domain search hit, but the function in replication or transcription was not certain. In addition, there were 18 ADRV proteins and 14 RGV proteins with unknown domains or functions. Among the 26 conserved proteins (core proteins) of iridoviruses, twenty were found among the identified proteins.

**Table 1:** Viral proteins identified by iPOND-MS analysis

Homologous proteins (ADRV-/RGV-)	Assays for ADRV			Assays for RGV			Possible function or contained domain	
	1	2	3	1	2	3		
							**Nucleotide precursor metabolism**	
22L/92R^a^		+	+				Thymidine kinase	
42R/73L^a^	+		+				Ribonucleotide reductase	
							**Replication**	
47L/63R^abc^	+	+	+	+	+	+	DNA polymerase: involved in the synthesis of polydeoxyribonucleotides	
85L/27R^bc^	+	+	+	+	+	+	SSB: binding single-stranded DNA	
88L/24R^abc^	+	+	+	+	+	+	D5 family NTPase: synthesis of RNA primers/unwinding double-stranded DNA	
23L/91R^abc^	+	+	+	+	+	+	Proliferating cell nuclear antigen: processivity factor for DNA polymerase	
							**Genome modification**	
24L/90R^bc^	+	+	+	+	+	+	Cytosine DNA methyltransferase: involved in genome DNA methylation	
							**DNA/RNA cleavage or specific structures processing**	
12L/102R^abc^	+	+	+	+	+	+	FEN endonuclease: involved in DNA replication, repair, or recombinantion	
10L/10L^abc^	+	+	+	+	+	+	DEAD-like helicase: involved in DNA replication or transcription	
28R/87L^ab^	+	+	+	+		+	Ribonuclease III	
50L/61R^b^	+			+	+	+	Putative Holliday junction resolvase: involved in DNA repair or recombination	
13R/101L^a^	+		+				Restriction endonuclease domain	
54R/56L				+		+	DEAD-like helicase: involved in DNA replication or transcription	
							**Transcription**	
9R/9R^abc^	+	+	+	+	+	+	DdRp II largest subunit, Rpb1	Components of RNA polymerase holoenzyme
46R/65L^abc^	+	+	+	+	+	+	DdRp II second largest subunit, Rpb2	
82L/30R	+						RNA polymerase Rpb5, C-terminal domain	
79L/34R^bc^	+	+	+	+	+	+	Transcription termination factor Rho domain	
							**Possessing domains but the functions in replication and transcription are not sure**	
68L/44R^abc^	+	+	+	+	+	+	RRV orf2-like protein	
83L/29R^abc^	+	+	+	+	+	+	Tyrosine kinase/lipopoly-saccride modifying enzyme	
91L/21R^abc^	+	+	+	+	+	+	2-cysteine adaptor domain; Protein kinase domain	
96L/16R^abc^	+	+	+	+	+	+	Putative AAA_ATPase	
29L/86R^bc^	+	+	+	+	+	+	Putative ATPase-dependent protease	
62R/50L^bc^	+	+	+	+	+	+	SAP domain	
19L/95R^ab^	+	+	+	+		+	Erv1/Alr family	
6R/6R^b^	+	+	+		+	+	US22 superfamily	
51L/60R^ab^	+		+		+	+	Putative phosphotransferase	
35R/80L	+	+	+				IBR domain	
26L/89R^b^	+				+		Immediate early protein ICP-18	
16L/98R^a^	+						Immediate early protein ICP-46	
65R/47L				+			104 kDa microneme/rhoptry antigen domain	
							**Unknown function/no putative conserved domains were found**	
3L/3L^bc^	+	+	+	+	+	+		
20R/94L^bc^	+	+	+	+	+	+		
61L/51R^bc^	+	+	+	+	+	+		
64R/48L^bc^	+	+	+	+	+	+		
97L/15R^b^	+	+	+	+		+		
98R/13L^ab^	+		+		+	+		
86L/26R^b^	+			+	+	+		
89R/23L^ab^	+			+	+	+		
38L/77R^b^	+			+		+		
5R/5R^b^	+		+	+				
45L/66R^b^	+			+		+		
94R/18L^b^	+					+		
66R/46L^b^	+				+			
100R/NA	+	+	+					
93R/19L		+	+					
63R/49L	+	+						
52L/59R	+							
4R/4R	+							
31R/84L				+				

“+” indicates the protein was detected in the corresponding iPOND assay

^a^The pair of proteins encoded by core genes of iridoviruses

^b^Proteins identified from both viruses infected samples

^c^Proteins detected in all assays

Furthermore, 20 of the 35 proteins were detected in all the experiments and were considered viral proteins with a high detection rate. These 20 proteins are listed in Fig. 2B by their abundances in MS analysis. For convenience, the following descriptions are focused on ADRV proteins. The proteins that had predicted functions in virus genome replication, transcription, repair, and modification included ADRV-47L (viral DPOL, vDPOL), ADRV-88L (D5 NTPase), ADRV-23L (viral proliferating cell nuclear antigen, vPCNA), ADRV-9R (viral RNAP II largest subunit, vPOL-IIα), ADRV-46R (viral RNAP II second largest subunit, vPOL-IIβ), ADRV-12L (DNA repair protein), ADRV-10L (DEAD helicase), ADRV-24L (cytosine DNA methyltransferase), and ADRV-79L (Rho domain containing protein). There were also proteins that could not be classified into the above categories, such as ADRV-20R.

### Identification and characterization of viral single-stranded DNA binding proteins

**Fig. 3:**
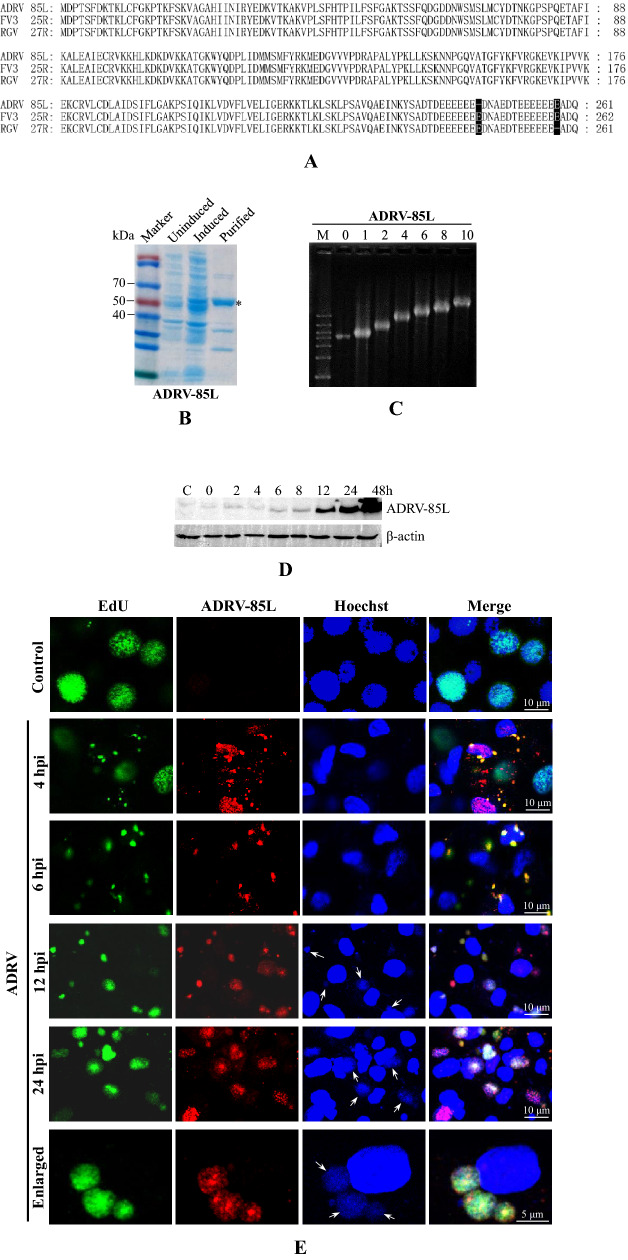
Characterization of ADRV-85L. **A** Multiple amino acid sequence alignment of ADRV-85L, RGV-27R, and FV3-25R. ADRV-85L and RGV-27R have two divergent sites (shaded in black) among their 261 aa. **B** Prokaryotic expression and purification of recombinant ADRV-85L. The protein markers, bacteria without induction (Uninduced), bacteria with induction (Induced), and purified proteins (Purified) are labeled at the top. The recombinant proteins with molecular weights of approximately 50 kDa are indicated with asterisks. **C** Electrophoretic mobility shift analysis of the DNA–protein complexes. The ΦX174 DNA–protein complexes migrated more slowly with increasing protein amounts (0‒10 μg). **D** Temporal expression of ADRV-85L in virus infected GSTC cells by Western blot analysis. **E** Subcellular localization of ADRV-85L in virus infected GSTC cells by immunofluorescence. Viral nascent DNA was labeled with EdU as described above (green). ADRV-85L was detected with anti-vSSB antibody (red). Cell nuclei were stained with Hoechst 33342 (blue). An enlarged photo shows the colocalized nascent DNA, ADRV-85L, and viral factories. Visible Hoechst-labeled cytoplasmic viral factories are indicated with arrows

When the protein abundances were analyzed, ADRV-85L showed high abundance among the obtained proteins, as did its homolog RGV-27R (Fig. 2B). Blast analysis showed that the two proteins had homologs in all sequenced ranaviruses, and the homolog in frog virus 3 (FV3 25R) was a previously identified early protein named P31K with unknown function [37]. Sequence alignment showed that the two proteins and P31K had high identities (Fig. 3A), with a difference in only one amino acid.

In view of the high abundances, the proteins should be essential components in DNA replication. *ADRV-85L* was then cloned and expressed in *E. coli*. The purified recombinant protein r85L containing the Trx-His tag had a molecular weight of approximately 50 kDa (Fig. 3B). In the subsequent electrophoretic mobility shift assay (EMSA), the migration rate of the single-stranded DNA of phage ΦX174 slowed with the addition of the recombinant protein in a dose-dependent manner (Fig. 3C). It revealed that the protein ADRV-85L is a viral single-stranded DNA binding protein (vSSB). RGV-27R was also expressed and purified. Similar results were obtained in the EMSA by using recombinant RGV-27R (Additional file 1: Fig. S4A–C).

The expression and localization of ADRV-85L were then examined by using an anti-vSSB antibody. Markedly enhanced bands for ADRV-85L were detected at 6 hpi by Western blot (Fig. 3D). There were weak bands at earlier times, which could be from the initial infected viral particles. In the subsequent immunofluorescence assay, EdU-labeled nascent DNA was detected in the cytoplasm at 4 hpi, and EdU labeling was colocalized with ADRV-85L. However, some of the cytoplasmic ADRV-85L was not localized with EdU labeling at this point. Some cell nuclei were labeled by the antibody, which showed a spotty pattern in the labeled nucleus. As the infection time increased, the size and intensity of the EdU-labeled cytoplasmic foci increased, and the amount of cytoplasmic ADRV-85L that did not colocalize was reduced. The number of nuclei that colocalized with ADRV-85L was also reduced. The colocalized EdU labeling and ADRV-85L were also colocalized with the Hoechst-labeled cytoplasmic viral DNA. At 24 hpi, the three colors displayed cytoplasmic punctate areas still colocalized (Fig. 3E). The expression and localization patterns of the vSSB protein between ADRV and RGV were similar, although RGV infection seemed more acute than ADRV infection (Additional file 1: Fig. S4D). Thus, vSSB, which is highly conserved in ranaviruses, was identified.

### Colocalization and interaction of replisome core components

In general, the essential components (replisome core components) needed for efficient DNA replication include helicase/primase, DPOL, SSB, and processivity factor. Among the 20 proteins with a high detection rate, ADRV-88L encodes a 975 aa protein that contains a primase domain at its N-terminus, a D5_N domain at the central region and an SF3 helicase domain at its C-terminus (Additional file 1: Fig. S5). It should be the virus-encoded helicase/primase (vHelicase/primase). ADRV-23L encodes a homolog of proliferating cell nuclear antigen (PCNA) which is the processivity factor for DPOL in eukaryotic cells. Therefore, the four components, the two described here plus vDPOL (ADRV-47L) (Additional file 1: Fig. S5) and vSSB (ADRV-85L) were found in the 20 proteins. The RGV homologs of ADRV-88L and ADRV-47L with the same sequence organizations are also shown in Additional file 1: Fig. S5.

**Fig. 4:**
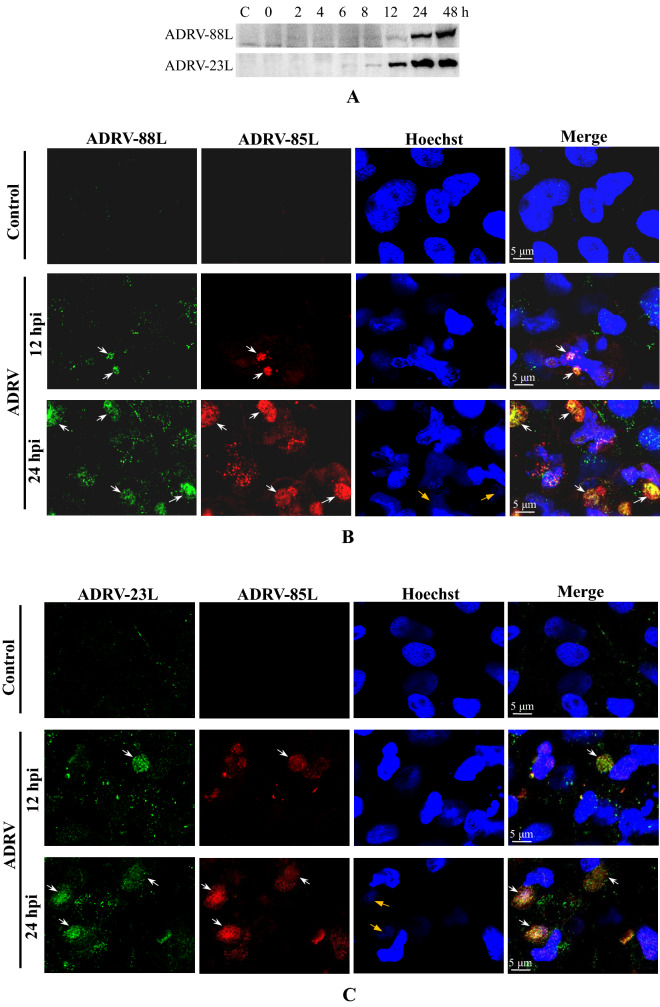

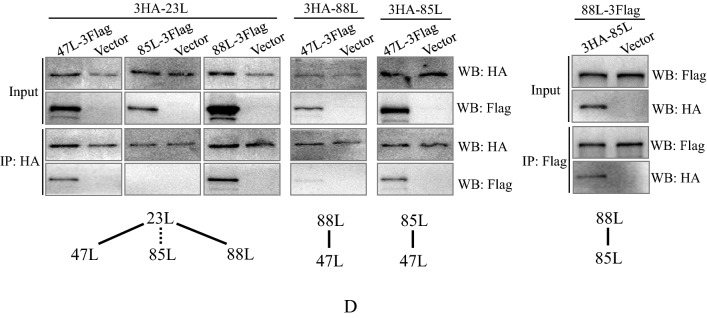
Temporal expression, localization, and interactions of replisome core proteins. **A** Western blot analysis of vHelicase/primase (ADRV-88L) and vPCNA (ADRV-23L). GSTC cells were infected by ADRV at 0.5 MOI and collected at indicated time points. **B** Localization of ADRV-88L by immunofluorescence in GSTC cells. ADRV-88L (green) was stained with an anti-88L antibody, and vSSB (red) was stained with an anti-vSSB antibody. Representative overlaps are indicated by white arrows. **C** Localization of ADRV-23L by immunofluorescence. ADRV-23L (green) was stained with an anti-23L antibody. Representative overlaps are indicated by white arrows. Visible Hoechst-labeled cytoplasmic viral factories are indicated with yellow arrows. **D** Interactions among the four replisome core proteins of ADRV (23L, 47L, 85L, and 88L) revealed by coimmunoprecipitations (co-IP). Cell lysates from HEK293T cells cotransfected with plasmids expressing the indicated proteins and immunoprecipitated (IP) protein complexes were subjected to Western blotting (WB) analysis using anti-HA or anti-Flag antibody. The interactions between two proteins are marked with black lines. A protein pair without interaction is indicated by a broken line

Polyclonal antibodies (anti-ADRV-88L and anti-ADRV-23L) were prepared using purified recombinant proteins. Expression of the two proteins, vHelicase/primase (ADRV-88L) and vPCNA (ADRV-23L), under viral infection was detected by Western blot. ADRV-23L was detected at 8 hpi. ADRV-88L was detected at 12 hpi, although weak bands could be observed at previous time points (Fig. 4A). Localization of the proteins in virus infections was then detected by immunofluorescence. The two proteins both localized in the cytoplasm in an aggregate state that colocalized with cytoplasmic factories, as indicated by the vSSB (Fig. 4B, C). The size and intensity of the stained proteins increased with infection time. Similar expression and localization patterns were obtained from RGV-infected cells (Additional file 1: Fig. S6).

Considering the colocalizations described above, the interaction relationships of the four replisome core proteins vDPOL, vHelicase/primase, vSSB, and vPCNA were investigated by coimmunoprecipitation (co-IP). The results showed that the fusion protein vDPOL (47L-3Flag) interacted with vPCNA (3HA-23L), vSSB (3HA-85L), and vHelicase/primase (3HA-88L), although the detected band between vHelicase/primase (3HA-88L) and vDPOL (47L-3Flag) was weak. The fusion protein vHelicase/primase (88L-3Flag) also interacted with vPCNA (3HA-23L) and vSSB (3HA-85L). Among pairs of the four proteins, only the interaction between vPCNA (3HA-23L) and vSSB (85L-3Flag) was not detected (Fig. 4D). The results indicated a complex interaction among the four proteins.

### Viral DPOL interacts with a number of viral proteins

**Fig. 5:**
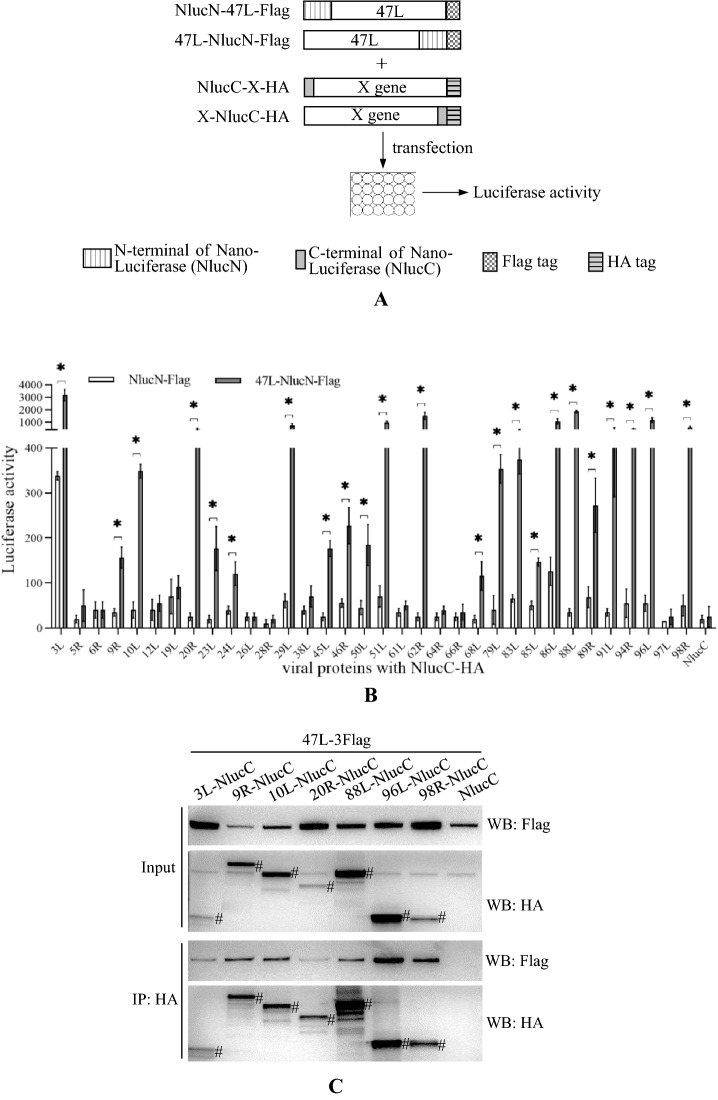

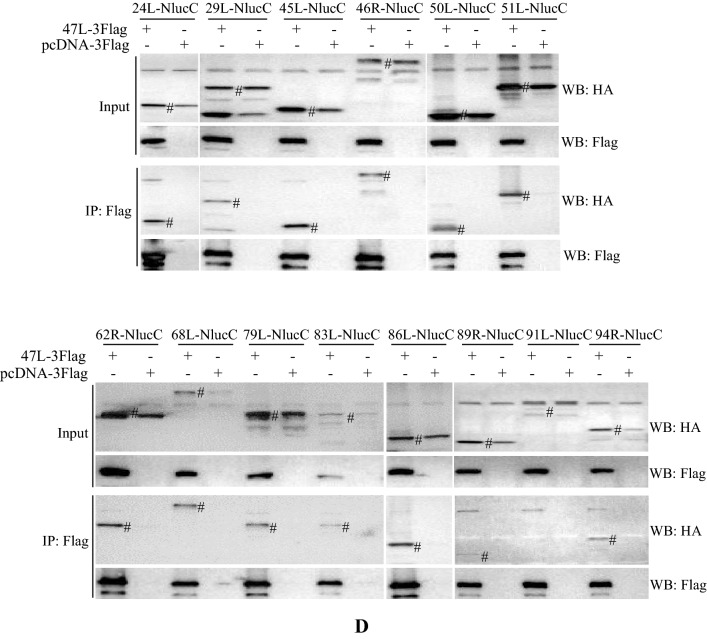
Screening and identification of viral proteins interacting with vDPOL (ADRV-47L). **A** Schematic diagram of the NanoLuc complementation assay in aquatic animal cells. The coding sequence of ADRV-47L was fused with N-terminus of NanoLuciferase (NlucN) in both orientation and Flag tag. Other genes under testing (X) were fused with the C-terminus of NanoLuciferase (NlucC) in both orientation and HA tag. Plasmids containing ADRV-47 and genes under testing were cotransfected into EPC cells. The luciferase activity was determined 48 h post transfection. The plasmid expressing NlucN was cotransfected with the genes under testing and used as a control. **B** NanoLuc complementation assay showed that twenty-three viral proteins (*p < 0.01) showed significantly higher luciferase activity in cells cotransfected with plasmids as described above. **C**, **D** Co-IP analysis of the protein interactions revealed by NanoLuc complementation assay. HEK293T cells were cotransfected with pcDNA-47L-3Flag and the plasmid expressing viral protein-NlucC-HA. The cells were then coimmunoprecipitated with anti-HA beads (**C**) or anti-Flag beads (**D**). The bands corresponding to the theoretical molecular weight of viral proteins are marked with #

The above results showed that vDPOL interacted with three viral proteins. We wondered whether vDPOL can interact with more viral proteins. The NanoLuciferase (NanoLuc, Nluc) complementation assay [38] was first used in aquatic animal cells to screen the interactions between ADRV-47L (vDPOL) and other viral proteins (Fig. 5A). The 34 ADRV proteins identified in both ADRV-infected and RGV (homologs)-infected samples except vDPOL itself were selected to perform the screening assay. The N-terminus of Nluc (NlucN, 18 kDa) was fused to vDPOL (47L) at its N- or C-terminus, while the C-terminus of Nluc (NlucC, 1.3 kDa) was fused to the viral proteins under test. The screening assay was performed in EPC and GSTC cells. The luciferase activity of several combinations showed a significant increase compared to their control. These combinations include 23 viral (ADRV) proteins: 3L, 9R, 10L, 20R, 23L, 24L, 29L, 45L, 46R, 50L, 51L, 62R, 68L, 79L, 83L, 85L, 86L, 88L, 89R, 91L, 94R, 96L, and 98R (Fig. 5B).

Co-IP was performed to further confirm the interactions between vDPOL and these proteins. To avoid the possible influence of the large tag NlucN on co-IP, the plasmid pcDNA-47L-3Flag expressing vDPOL with a 3× Flag tag was used. Proteins 23L and 85L were not selected in the assay because their interactions with vDPOL were identified in the above results. A total of 20 proteins were expressed successfully in HEK293T cells, while 91L was not. Although the expression levels of the proteins were not identical, the positive bands (anti-Flag bands in Fig. 5C and anti-HA bands in Fig. 5D) were all detected from coimmunoprecipitated (IP) protein complexes. The Nluc complementation assay and co-IP both confirmed that the viral replisome core protein vDPOL interacts with a number of viral proteins.

### Host topoisomerases IIα and IIβ are core viral replisome components

Hundreds of host proteins were identified in the captured protein mixtures by MS. However, most of the identified proteins have functions related to chaperones, ribosomes, translation initiation and elongation. The present study focused on the proteins participating in viral genome replication and transcription, so only the proteins with definite or potential functions in replication and transcription were chosen (Table 2). Among them, DNA topoisomerase IIα (TopIIα) was identified in both virus-infected samples with high abundance, and topoisomerase IIβ (TopIIβ) was identified in RGV samples.

**Table 2:** Host proteins identified by iPOND-MS analysis

Proteins	Assays for ADRV			Assays for RGV			Possible functions
	1	2	3	1	2	3	
Topisomerase IIα	+	+	+	+		+	DNA topological change
Topisomerase IIβ				+		+	
FACT complex subunit SSRP1	+	+	+			+	DNA replication, DNA repair, and mRNA elongation
FACT complex subunit SPT16	+	+	+			+	
Transcriptional repressor p66-beta	+	+		+	+		Transcriptional repressor
Histone H2A.x-like			+	+	+	+	DNA repair
Heterogeneous nuclear ribonucleoprotein A1	+					+	RNA binding/processing
ATP-dependent RNA helicase A		+	+		+		RNA helicase
ATP-dependent RNA helicase DDX3X			+				RNA helicase
RNA polymerase II subunit Rpb3-like					+	+	Transcription
DNA ligase 3				+			DNA repair

“+” indicates the protein was detected in the corresponding iPOND assay

**Fig. 6:**
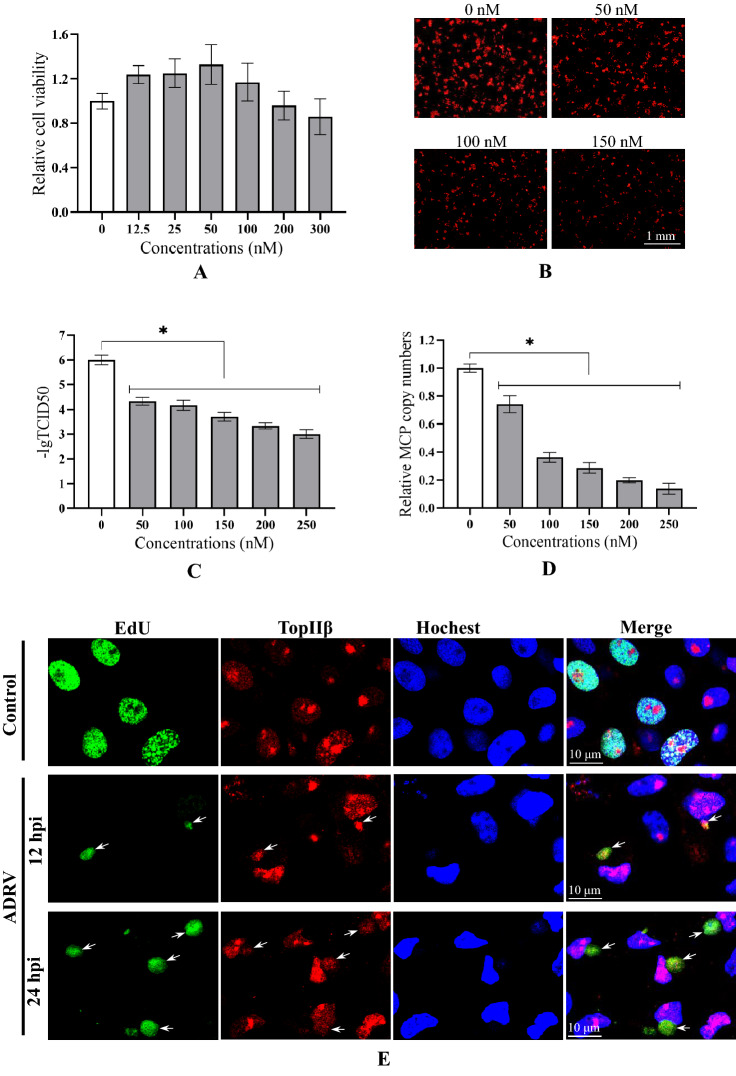

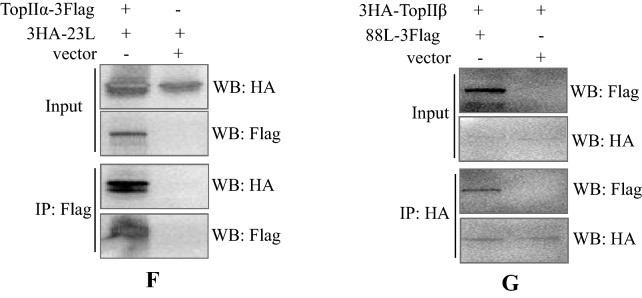
Functional analysis of host TopIIα and TopIIβ in ranavirus infection. **A** Cell viability of GSTC after doxorubicin treatment for 24 h. **B** Observation of virus infection after doxorubicin treatment. GSTC cells treated with doxorubicin were infected with recombinant ADRV expressing mCherry and observed at 24 hpi. **C** Determination of virus titers after drug treatment. The virus titers were determined by TCID_50_ methods, which showed that ADRV titers were reduced by drug treatment. **D** Detecting viral relative genome copy numbers by qPCR. The relative genome copy numbers were determined by calculating the relative MCP copy numbers. Significant differences (versus virus without drug) are marked with *(p < 0.05). **E** Subcellular localization of host TopIIβ in ADRV-infected GSTC cells by immunofluorescence. Cytoplasmic TopIIβ (red) was colocalized with EdU labeling (green). Representative merges were indicated by white arrows. **F** Detection of the interaction between TopIIα and ADRV-23L by co-IP. G. Detection of the interaction between TopIIβ and ADRV-88L by co-IP. Cell lysates from HEK293T cells cotransfected with plasmids expressing the indicated proteins and immunoprecipitated (IP) protein complexes were subjected to Western blot (WB) analysis using anti-HA or anti-Flag antibody

Because of their detected high abundances and importance in genome replication, DNA TopIIα and TopIIβ were further investigated. We first used a topoisomerase II inhibitor, doxorubicin, to investigate their function in virus replication and infection. When a recombinant ADRV expressing an mCherry tag in the TK locus was used, the results showed that the inhibitor drug significantly reduced virus infection at noncytotoxic concentrations (Fig. 6A, B), as proven by TCID50 determination (Fig. 6C). Further qPCR detection also showed that the relative amount of virus genomes decreased with increasing drug concentrations (Fig. 6D). Therefore, the host TopIIα and TopIIβ are needed in ADRV infection.

To further verify the involvement of host topoisomerase II in ranavirus infection, a commercial anti-topoisomerase II antibody (Abcam, ab109524, immunogen of the antibody was declared as a fraction of the human TopIIβ) was used based on the high homology of the protein among different species. Fortunately, the antibody could recognize a specific band corresponding to TopIIβ expression in GSTC cells in western blotting analysis. TopIIβ localization was then investigated by immunofluorescence. In GSTC cells without infection, TopIIβ was localized in the nuclei, and some of the nuclei were stained by EdU. In ADRV-infected cells, TopIIβ appeared in the cytoplasm and colocalized with EdU labeling. The size and intensity of the cytoplasmic labeling increased with the infection time (Fig. 6E). This result revealed that the host TopIIβ can transfer to the cytoplasm and colocalize with virus nascent DNA during infection.

To further investigate the viral proteins that interact with host TopIIα and TopIIβ, coding sequences of TopIIα and TopIIβ were cloned from GSTC cells. The open reading frame of TopIIα is 4665 bp in length, which encodes a protein containing 1554 amino acids (aa), while TopIIβ is 5145 bp in length, encoding a protein with 1714 aa. The sequences have been deposited into GenBank under accession numbers MW465413 and MW465414. In the following co-IP assays, the fusion protein 3HA-23L (vPCNA) was coimmunoprecipitated with TopIIα-3Flag (Fig. 6F), and 88L-3Flag (vHelicase/primase) was coimmunoprecipitated with the 3HA-TopIIβ (Fig. 6G), which showed their interactions. The above results indicate that host TopIIα and TopIIβ are recruited by viral replisome core proteins to constitute the viral replisome.

### Identification of proteins associated with viral transcription machinery

**Fig. 7:**
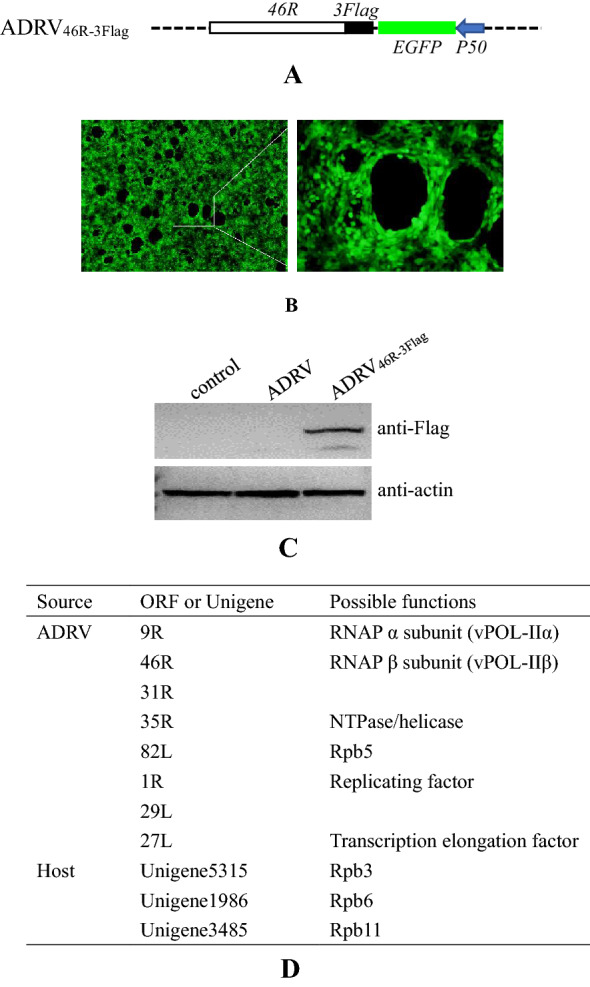
Identification of ADRV transcription machinery related proteins with an ADRV_46R-3Flag_ recombinant virus. **A** Construction of recombinant ADRV_46R-3Flag_ expressing the 46R-3Flag fusion protein. A 3× Flag tag was fused to the C-terminus of 46R. EGFP driven by the viral P50 promoter was used as a selection marker. The dashed line indicates the virus genome. **B** Fluorescence micrograph of recombinant virus infected cells showing the expression of EGFP. **C** Western blotting analysis of recombinant virus infected cells with anti-Flag antibody. 46R-3Flag expressed successfully. **D** Viral and host proteins identified by MS analysis

Some proteins related to virus transcription were revealed by the iPOND assay, but the number was not enough to constitute a transcriptional machinery, and they need to be confirmed by other methods. To further investigate the virus transcription machinery, we tried to construct recombinant viruses possessing Flag-tagged RNA polymerase. The recombinant virus ADRV_46R-3Flag_, which had a 3× Flag tag fused to the C-terminus of 46R (homolog of RNAP II β subunit, vPOL-IIβ), was constructed and purified successfully (Fig. 7A–C). By affinity purification of proteins associated with 46R-3Flag, 8 viral proteins and 3 host proteins were identified (Fig. 7D). The 8 viral proteins include 9R (homolog of RNAP II α subunit, vPOL-IIα), 46R, 31R (possible transcription factor), 35R, 82L, 1R (replicating factor), 29L, and 27L (transcription elongation factor), among which the 9R, 46R, 31R, 35R, 82L, and 29L were also identified in the iPOND-MS assay. The 3 host proteins were the host RNAP II subunits Rpb3, Rpb6, and Rpb11.

**Fig. 8:**
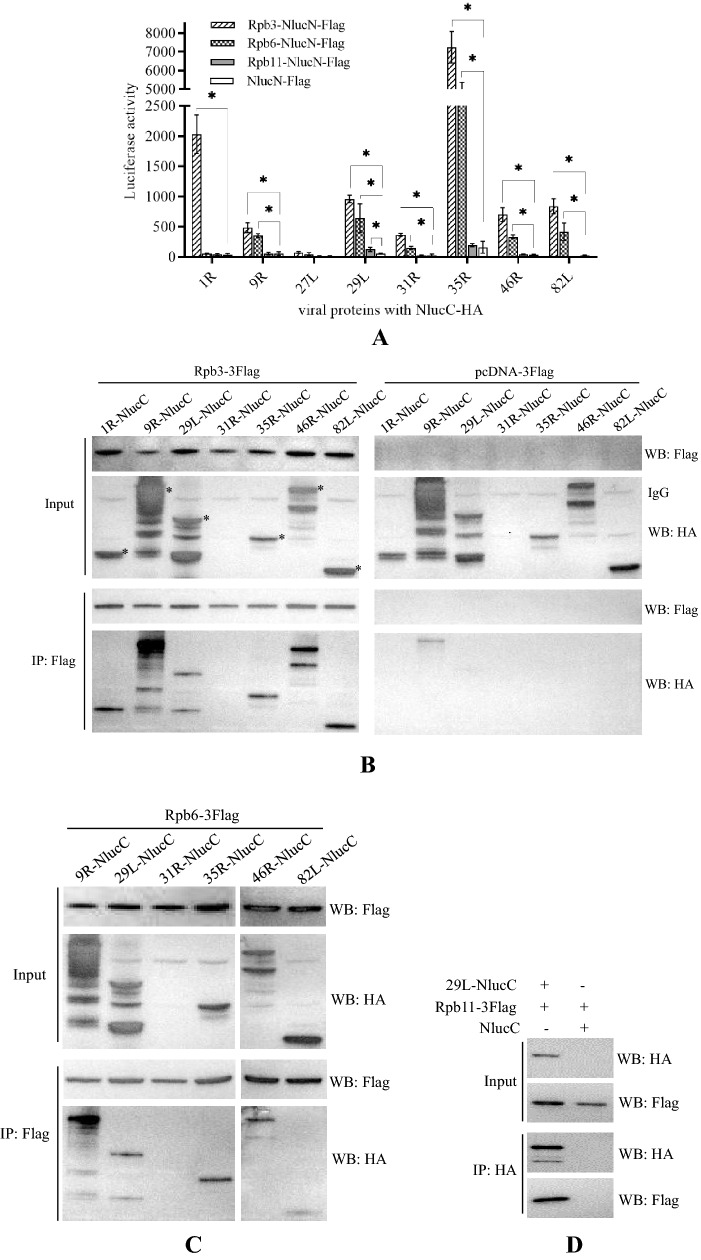
Identification of the interactions between ADRV proteins and host Rpb3, Rpb6, and Rpb11. **A** NanoLuc complementation analysis of the interactions. A significant increase (*p < 0.05) in luciferase activity was observed between Rpb3 and seven viral proteins, Rpb6 and six viral proteins, and Rpb11 and one viral protein. **B** Co-IP analysis of the interactions in HEK293T cells. Cells were cotransfected with plasmid pcDNA-Rpb3-3Flag and the plasmid expressing viral proteins with NlucC and HA, and coimmunoprecipitated with anti-Flag beads (left panel). The plasmid pcDNA-3Flag was used as a control (right panel). The bands corresponding to viral proteins are indicated by asterisks. **C** Co-IP analysis of the interactions in HEK293T cells. Cells were cotransfected with plasmid pcDNA-Rpb6-3Flag and the viral gene containing plasmids above, and coimmunoprecipitated with anti-Flag beads. **D** Co-IP analysis of the interactions in HEK293T cells. Cells were cotransfected with plasmids pcDNA-Rpb11-3Flag and pcDNA-29L-NlucC-HA, and coimmunoprecipitated with anti-HA beads

If host Rpb3, Rpb6, and Rpb11 are involved in viral transcription, they should interact with viral proteins. We then cloned *Rpb3/Rpb6/Rpb11* (GenBank accession number: MW558047, MW558048, and MW558049) and performed a NanoLuc complementation assay and co-IP analysis to investigate the interactions. In the NanoLuc complementation assay, a significant increase in luciferase activity was observed between Rpb3 and 1R, 9R, 29L, 31R, 35R, 46R, and 82L. A significant increase was also observed between Rpb6 and 9R, 29L, 31R, 35R, 46R, and 82L. For Rpb11, a significant increase was observed only from 29L (Fig. 8A).

In co-IP analysis, 1R-NlucC-HA, 9R-NlucC-HA, 29L-NlucC-HA, 35R-NlucC-HA, 46R-NlucC-HA, and 82L-NlucC-HA all coimmunoprecipitated with Rpb3-3Flag (Fig. 8B), indicating their interactions. The expression of 31R-NlucC-HA was not detected in HEK293T cells. In the control group, which used the plasmid pcDNA-3Flag instead of pcDNA-Rpb3-3Flag, no obvious bands were observed in most of the coimmunoprecipitated protein complexes, but a weak band for 9R-NlucC-HA appeared, which could result from the high expression efficiency of the gene in the cells. Similar to the results from Rpb3, 9R-NlucC-HA, 29L-NlucC-HA, 35R-NlucC-HA, 46R-NlucC-HA, and 82L-NlucC-HA were all coimmunoprecipitated with Rpb6-3Flag (Fig. 8C). Rpb11-3Flag was coimmunoprecipitated with 29L-NlucC-HA (Fig. 8D).

### Host Rpb3, Rpb6, and Rpb11 were needed in ADRV infection in BHK-21 cells

To further verify the function of host Rpb3, Rpb6, and Rpb11 in ADRV infection, we used baby hamster kidney fibroblast cells (BHK-21) to perform immunofluorescence and RNA interference (RNAi) assay for the availability of commercial antibodies and sensitivity to the virus. BHK-21 cells have been used in research on frog virus 3 [39] and their sensitivity to ADRV and RGV was also proven in our preliminary experiment.

**Fig. 9:**
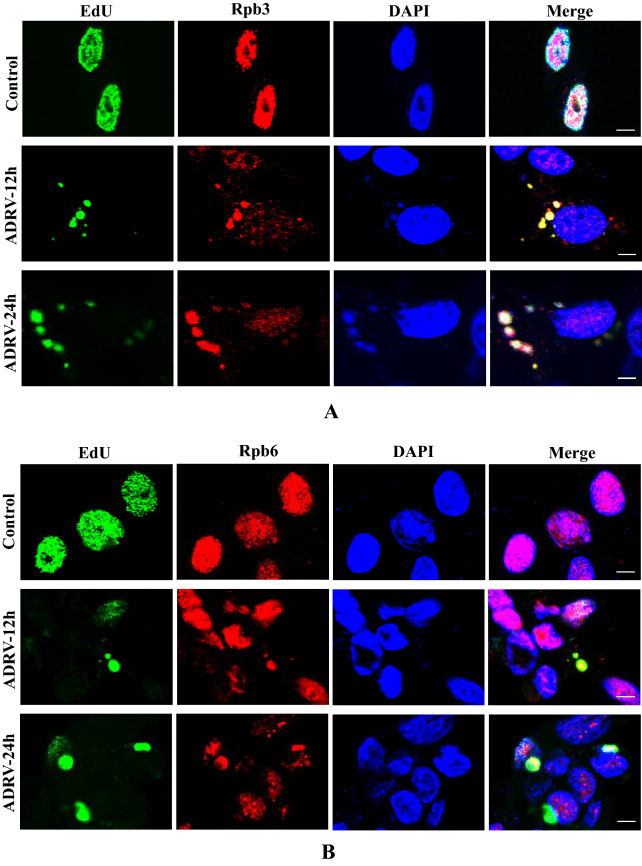

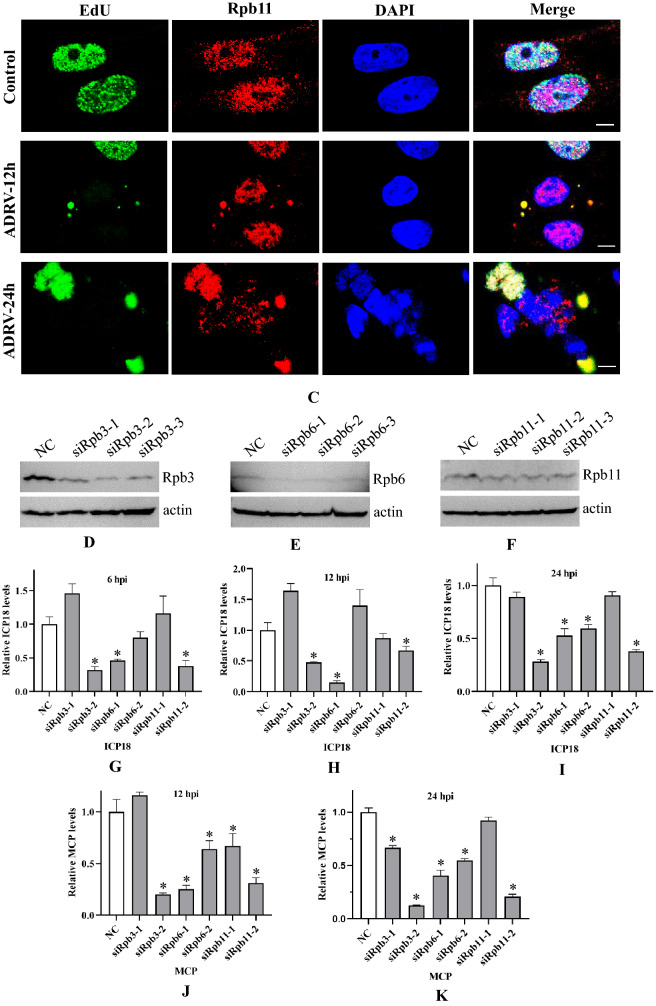
Host Rpb3, Rpb6, and Rpb11 were involved in ADRV infection in BHK-21 cells. **A**–**C** Localization of Rpb3 (**A**), Rpb6 (**B**), and Rpb11 (**C**) in viral factories in ADRV infected BHK-21 cells. The cells were infected with or without ADRV at an MOI of 1 and fixed at the indicated time points. Rpb3, Rpb6, and Rpb11 (red) were stained with commercial antibodies. Replicating DNA was stained with EdU (green) as described above. Cell nuclei were stained with DAPI (blue). Bar = 5 μM. **D**–**K** Inhibition of the expression of *Rpb3*, *Rpb6*, and *Rpb11* reduced ADRV gene expression in BHK-21 cells. siRNAs targeting *Rpb3*, *Rpb6*, and *Rpb11* were transfected into the cells. The transfected cells were subjected to western blotting analysis (**D**–**F**) or ADRV infection. Expression of the ICP18 gene (**G**–**I**) and MCP gene (**J**, **K**) at different time points was determined by RT–qPCR

Rpb3, Rpb6, and Rpb11 in normal BHK-21 cells without virus infection were distributed mainly in the cell nuclei. However, the three proteins all appeared in the cytoplasm and colocalized with EdU-labeled viral factories during ADRV infection. The colocalization became more obvious as the infection time increased (Fig. 9A–C). A similar phenomenon was observed in RGV-infected BHK-21 cells (Additional file 1: Fig. S7).

We further performed RNAi in BHK-21 cells. Among the three siRNAs for each gene, siRpb3-2, siRpb6-1, and siRpb11-2 possessed the highest inhibition efficiency for the expression of their targeted genes, as revealed by western blot analysis (Fig. 9D–F). Then, two siRNAs for each gene, including the one having the highest inhibition efficiency, were used for further analysis. RT–qPCR showed that the three siRNAs (siRpb3-2, siRpb6-1, and siRpb11-2) all significantly reduced the expression of the ADRV immediate early gene *ICP18* and late gene *MCP* at the detected time points compared to the control NC (Fig. 9G–K). Compared to the three siRNAs, the other siRNAs (siRpb3-1, siRpb6-2, siRpb11-1) showed a weak inhibitory effect, which was in line with the inhibition efficiency revealed by western blot. Thus, subcellular localization and RNAi assays both revealed that host Rpb3, Rpb6, and Rpb11 participate in ADRV transcription.

## Discussion

Ranaviruses are becoming a global threat to wild or economically important lower vertebrates, while the mechanism of their replication and transcription remains largely unresolved. In the present study, we used two ranaviruses (ADRV and RGV) to explore ranavirus replication and transcription. The number of the identified proteins from ADRV- and RGV-infected samples showed a little difference in iPOND-MS, which could result from the different infection rates between the two viruses. qPCR in the present study showed that RGV started genome replication earlier than ADRV. Our previous study also showed that RGV replicated faster than ADRV and induced different host responses in vivo [40]. The difference in infectious behaviour could lead to differences in protein abundances when the samples were collected at the same time. To elucidate the common characteristics of ranavirus replication and transcription, the present study focused on the common proteins shared by the two viruses and verified them mainly in ADRV-infected cells.

The present study established a protocol for EdU labeling in ranavirus infected aquatic animal cells. The EdU-labeled nascent DNA during ADRV or RGV infection was observed in the cytoplasm in the assays. However, it has been reported that ranavirus replication takes place in two stages. In the first stage, the unit length genomes are synthesized in the nucleus [41]. The lack of observed EdU labeling of viral genome in the nucleus could result from two reasons. The first is that the weak signals emitted from the nuclear viral unit length genome might have been swamped by the large excess of host DNA. The other was that the time point of EdU addition might have been later for the first stage of virus replication. The exact reason needs to be explored in future.

One interesting finding in the present study is that vDPOL (ADRV-47L) interacts with a series of viral proteins. Although vDPOL is one of the core components in DNA replication, its interactions with so many viral proteins have rarely been reported in DNA viruses. The interacting proteins with predicted functions included not only vPCNA, vSSB, and vHelicase/primase, which function as core proteins in DNA replication, but also vPOL-IIα (ADRV-9R) and vPOL-IIβ (ADRV-46R), which function as core enzymes in DNA transcription. ADRV-24L, a virus encoding DNA methyltransferase, also interacted with vDPOL. The interacting proteins included the proteins involved in DNA replication, modification, and transcription. It seems that vDPOL not only is the key enzyme in viral DNA replication but also has regulatory function in viral DNA-related events.

Current data show that the replisome core proteins of ADRV and RGV contain the following components: vDPOL (ADRV-47L/RGV-63R), vHelicase/primase (ADRV-88L/RGV-24R), vPCNA (ADRV-23L/RGV-91R), and vSSB (ADRV-85L/RGV-27R). Generally, the core enzymes/proteins in DNA replication include a helicase, primase, DPOL, processivity factor, clamp loader, and SSB [19]. ADRV-88L (or its homolog RGV-24R) contains a primase domain at its N-terminus, a D5_N domain at the central region and an SF3 helicase domain at its C-terminus. The D5 domain comes from the VACV D5R protein, which is a 95 kDa protein containing an N-terminal primase domain and a C-terminal SF3 helicase domain and has been identified as a virus-encoded helicase-primase fusion protein [42, 43]. Thus, ADRV-88L and RGV-24R should be viral helicase/primase. ADRV-23L and RGV-91R (98.0% sequence identity) are homologs of the eukaryotic PCNA. PCNA has been identified as the “sliding clamp” processive factor of DPOL in eukaryotes [44]. The DNA replication processive factor is different in different NCLDVs. For example, the African swine fever virus encodes a PCNA homolog [45], but VACV encodes two proteins that function as processive factor [46, 47]. The SSB of ranaviruses cannot be predicted by BLAST search. However, the protein was observed in the iPOND-MS data with high abundance and was further characterized. As an essential component in DNA replication, SSB binds to single-stranded DNA to make it resistant to nucleases and remove barriers of intramolecular secondary structures [48]. We further analyzed the interaction relationships among the four proteins, which showed complex and interesting interactions among the proteins except that between vSSB and vPCNA. DNA replication involves a series of cooperative reactions that occur in the replication fork, such as DNA unwinding, RNA primer synthesis, leading strand synthesis, lagging strand synthesis, SSB protein binding and removal, RNA primer removal. The interactions, including DPOL-PCNA, DPOL-primase, DPOL-SSB, and primase-SSB, which have been reported in other organisms [49–52], would benefit cooperative reactions in ranavirus DNA replication. The comprehensive interactions among the four proteins also support their roles as replisome core proteins of ranaviruses.

For large DNA viruses, genomic DNA replication is usually coupled with transcription processes [22], which enables transcription-related proteins to be identified from viral nascent DNA-associated proteins [31, 34]. In the present study, some proteins involved in transcription were found in the iPOND-MS data. For the virus-encoded proteins, two RNAP II subunits, vPOL-IIα and vPOL-IIβ, were revealed with high detection rates, which were also belonged to the core genes of iridoviruses. RNAP II is a multisubunit enzyme that is conserved in eukaryotes and needs several factors, including transcription factors, to initiate and achieve efficient transcription [53]. Interestingly, a previously unnoted viral protein, ADRV-82L, was identified in our iPOND data and found to contain an RNAP subunit Rpb5 domain. Interestingly, among the host proteins, an Rpb3 subunit was found in RGV-infected samples. In addition to the RNAP subunits, a previously reported serine/threonine kinase, ADRV-79L/RGV-34R, was found to contain a transcription termination factor Rho domain in its N-terminal region. Overall, some proteins related to viral DNA transcription were identified by iPOND-MS assay, but there could be other transcription-related factors in the identified proteins due to low protein homology. A previous report predicted 3 RNAP subunit orthologs, vPOL-IIα, vPOL-IIβ, and transcription elongation factor TFIIS, in ranavirus-encoded proteins [54], which hinted that ranaviruses could not encode complete RNAP subunit orthologs. Moreover, the TFIIS ortholog was not found in the iPOND data.

To further investigate the proteins involved in virus DNA transcription and verify the iPOND data, we tried to add a tag to virus RNA polymerase to perform affinity purification. Recombinant ADRVs expressing 46R-6His, 46R-10His, and 46R-3Flag were constructed and purified. Flag-based affinity purification was finally used to identify viral RNAP-related proteins for the mild purification conditions. The obtained ADRV proteins included six (9R, 46R, 31R, 35R, 82L, and 29L) that were observed in the iPOND assay, including the predicted Rpb5 (82L), and two (1R and 27L) newly identified proteins, which were predicted to be ranavirus replication factors and TFIIS, respectively. ADRV-35R was predicted to be an NTPase/helicase-like protein, and ADRV-29L was predicted to be an ATPase-dependent protease in previous ranavirus genome annotations [6, 55]. No putative conserved domains could be found in ADRV-31R. However, by PSI-BLAST, ADRV-31R was found to have sequence similarity with transcription factors in some viruses, such as the VLTF2-like transcription factor in poxvirus. Among the host proteins, the RNAP II subunits Rpb3, Rpb6, and Rpb11 were identified and further proven to be involved in ADRV gene transcription. In particular, Rpb3 interacted with 7 viral proteins, and Rpb6 interacted with 6 viral proteins. Previous reports have shown that Rpb3 is an anchoring center for other Rpb subunits [56, 57]. Therefore, it can be speculated that ranavirus (ADRV) recruits host RNAP subunit Rpb3/Rpb6/Rpb11 to constitute a hybrid RNAP to complete its gene transcription. No such hybrid RNAP was reported in other NCLDVs. It seems that the host Rpb3 also has an anchoring center function in the hybrid transcription machinery.

Efficient genomic DNA replication and transcription requires the cooperation of a series of enzymes or proteins. In addition to the core proteins described above, there are still other important participants. For example, FEN nucleases are critical in DNA repair and recombination for their exo/endonuclease activities [58]. In the present study, virus-encoded FEN1-like proteins (ADRV-12L/RGV-102R) were identified in the iPOND-MS analysis and were detected in all six samples. Deletion of ADRV-12L impaired virus infection (unpublished observations by Ke et al.). Topoisomerases alter the supercoiling of double-stranded DNA caused by the compaction of DNA and the continuous action of DNA transactions, including replication, recombination, and transcription [59]. Ranaviruses do not encode a homolog of topoisomerase but utilize host topoisomerase II. In our iPOND-MS assays, TopIIβ was obtained only in RGV samples. This could result from the relatively high protein abundances in viral factories caused by the high infection rate of RGV. Our further immunofluorescence and co-IP both proved the involvement of host TopIIβ in ADRV replication. DNA ligases have important functions in DNA replication, repair, and recombination [60]. It has been reported that VACV encodes a DNA ligase homolog [61], but no such homolog was found in ranaviruses. As expected, a homolog of DNA ligase 3 (DL3) was found with low abundance among the host proteins of an RGV-infected sample. It could be speculated that the viral FEN-1-like protein and host DNA ligase 3 have functions in primer removal and ligation of the Okazaki fragments on the lagging strand in ranavirus DNA replication. In addition, the virus-encoded cytosine DNA methyltransferase had a high detection rate, which was consistent with a previous report that the ranavirus genome was methylated after its replication [62].

It is worth noting that there are seven species in the genus *Ranavirus*, as revealed by the ICTV report [7]. Most of the species have high protein sequence identities, such as more than 90% sequence identity for the MCP. ADRV and RGV belong to two species, the common midwife toad virus and frog virus 3, respectively. Because of the high similarity in genome size, gene content, and protein sequence in most of the species, it could be speculated that the replication and transcription mechanisms obtained from our study will apply to most of the ranavirus isolates. However, these isolates that showed distinct differences in gene content and protein sequence identity could have unique replication and transcription characteristics.

## Conclusions

**Fig. 10:**
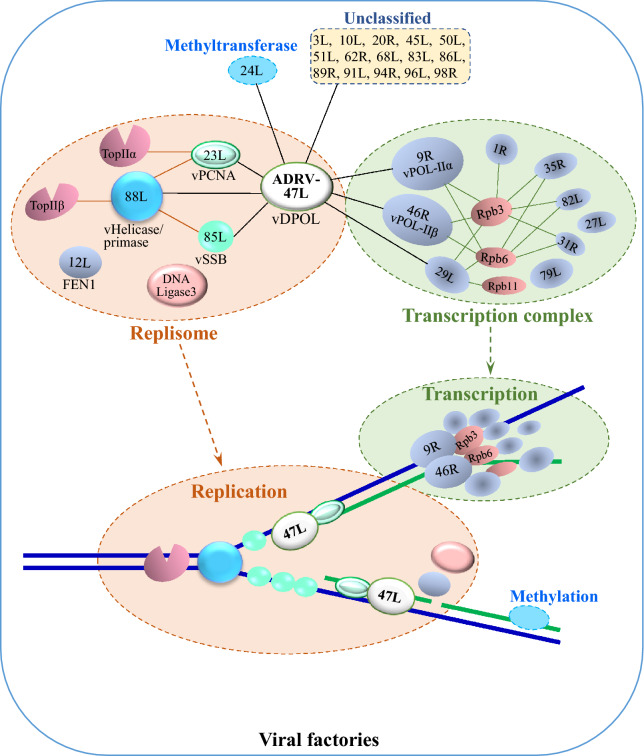
Schematic diagram of ranaviral (ADRV) replication and transcription machinery in viral factories. Proteins that interacted directly with ADRV-47L are indicated by thin black lines, and the other interactions are indicated by thin pink or green lines. The viral DNA strands are indicated by thick line with blue and green (nascent) color. The dashed box includes a group of protein components with the corresponding functions: Replisome (8 proteins), Transcription complex (12 proteins), Methylation (1 protein), and unclassified (15 proteins). Viral proteins are indicated by blue or green cake, and host proteins are indicated by pink cake

In summary, our above results have uncovered the complicated replication and transcription machinery of ranaviruses for the first time by using two ranaviruses. The machinery includes at least 30 viral proteins and 6 host proteins with a rarely reported vDPOL core (Fig. 10). Meanwhile, we identified the ranavirus SSB, revealed the essential roles of several host proteins, and deeply dissected the interactions and correlations among components in the machinery of the replisome and transcription complex, which enable efficient coordination of viral DNA replication, modification and transcription. Therefore, the current study opens a window of insight into understanding the efficient replication and transcription of ranaviruses, and for further comprehending the interactions between ranaviruses and their hosts.

## Materials and methods

### Viruses and cell lines

ADRV and RGV that were maintained in our laboratory were used in the present study. The Chinese giant salamander thymus cell (GSTC) line and the *Epithelioma papulosum cyprini* (EPC) cell line were cultured in M199 medium supplemented with 10% bovine calf serum at 25 °C until use. Baby hamster kidney fibroblast cells (BHK-21) and human embryonic kidney (HEK293T) cells were grown at 37 °C in 5% CO_2_ in Dulbecco’s modified Eagle’s medium (DMEM) supplemented with 10% bovine calf serum.

### EdU labeling and confocal microscopy

GSTC cells or BHK-21 cells plated on coverslips were mock infected or infected with ADRV or RGV at an MOI of 1. After 1 h of adsorption at 25 °C, the inoculum was removed, and the cells were washed two times with fresh medium. The infected cells were then cultured at 25 °C with fresh medium. At the times indicated, EdU (Invitrogen) was added to a final concentration of 10 μM. After continued incubation for the indicated times in each experiment, the cells were washed with phosphate-buffered saline (PBS), fixed with 4% paraformaldehyde for 20 min at room temperature, and then processed with a Click-iT Plus EdU Cell Proliferation Kit for Imaging (Thermo Scientific) according to the manufacturer’s protocols. Briefly, the cells were permeabilized with 0.5% Triton X-100 for 20 min at room temperature and subjected to a click reaction in a buffer provided in the kit containing Alexa Fluor 488-azide. In some cases, the cells were then incubated with antibodies against viral or host proteins (rabbit anti-RGV-27R, prepared in the study; rabbit anti-topoisomerase II, Abcam, ab109524; rabbit anti-Rpb3, ABclonal, A1785; rabbit anti-Rpb6, Proteintech, 15334-1-AP; rabbit anti-Rpb11, Proteintech, 16403-1-AP), followed by the corresponding secondary antibody conjugated to Alexa Fluor 546. Finally, the cells were stained with Hoechst 33342 or DAPI, and coverslips were mounted with ProLong Gold (Thermo Scientific). Images were collected on a Leica TCS SP8 confocal microscope.

### Detection of virus genome amplification

GSTC cells seeded in 24-well plates were infected with ADRV or RGV at an MOI of 0.1. After 1 h of incubation at 25 °C, the inoculum was removed, and the cells were washed three times with fresh medium. The infected cells were then cultured with fresh medium at 25 °C. The samples were collected at the indicated time points. DNA was extracted from the cells with a TakaRa MiniBEST Universal Genomic DNA Extraction Kit (TakaRa, Japan). Viral major capsid protein gene (MCP) copy numbers were detected by quantitative real-time PCR (qPCR), which was conducted using a StepOne Real-Time PCR system (Applied Biosystems, USA) as described previously [63]. The plasmid pMD18T-MCP, which was constructed by inserting the MCP product into the pMD18-T vector, was used as a template to construct the standard curve. The MCP amount (genome copy number) of each sample was calculated based on the standard curve.

### iPOND

The iPOND experiment was performed according to protocols described previously [30]. For each iPOND experiment, eight 75 cm^2^ cell culture flasks containing 2 × 10^8^ GSTC cells were infected with ADRV or RGV at an MOI of 3. After 1 h of adsorption at 25 °C, the infection medium was removed, and the cells were washed two times with fresh medium. The cells were then cultured for 7 h at 25 °C. EdU was added at a final concentration of 10 μM, and the cells were incubated for another 30 min. The cells were fixed with 1% formaldehyde and incubated for 20 min at room temperature and then quenched with 0.125 M glycine. After washing with PBS, the cells were scraped, collected, and permeabilized with 0.25% Triton X-100 for 30 min at room temperature. The cells were washed once with cold 0.5% BSA in PBS and once with cold PBS. Before the click reaction, the cells were divided into two pieces for experimental and control samples. The click reaction reagents were added in the following order to the following concentrations in 5 ml: biotin azide, 10 μM; sodium ascorbate, 10 mM; and CuSO_4_, 2 mM. For the control sample, DMSO was added instead of biotin azide. The reactions were performed at room temperature for 2 h. The cells were washed once with cold 0.5% BSA in PBS, then once with cold PBS, and resuspended in lysis buffer (50 mM Tris, pH 8.0; 1% SDS) containing protease inhibitor cocktail (Promega). The cells were sonicated by a Covaris S2 System and centrifuged at 16,100×*g* for 10 min. The DNA fragmentation size (Additional file 1: Fig. S8) was examined with a method described in reference [30]. After centrifugation, the supernatant was collected and diluted 1:1 with cold PBS containing protease inhibitor cocktail. Capture of DNA–protein complexes was carried out by incubating the diluted mixture with streptavidin-agarose beads (Novagen) for 16–20 h at 4 °C with rotation. The beads were washed once in cold lysis buffer, once in 1 M NaCl, and two times in lysis buffer. Finally, the proteins were eluted in 4% SDS loading buffer at 95 °C for 25 min. The resulting isolates were separated on ~ 1 cm on a 12% SDS–PAGE gel, excised into four pieces and analyzed by mass spectrometry.

### Mass spectrometry

The gel pieces were processed with standard mass spectrometry protocols. The tryptic digests were analyzed in an Easy-nano liquid chromatography system (Thermo Scientific) equipped with an Easy-Spray 15-cm column (C18, 2 μm, 75 μm × 15 cm; Thermo Scientific) coupled to an LTQ Orbitrap Elite mass spectrometer (Thermo Scientific) as described previously [64].

High-resolution mass spectra of the peptide mixture were deconvoluted using Xtract software (Thermo Scientific). The analysis of the mass spectrometric RAW data was carried out using Proteome Discoverer 2.1. The coding products from predicted ORFs of ADRV (KC865735.1) and RGV (JQ654586.1) and the products of the unigenes from transcriptomes of Chinese giant salamander (SRP115981) were used as the search database [6, 40, 55]. Three independent iPOND experiments containing click and nonclick controls were performed for each virus. The positive identification of a protein followed the following criteria: the protein was present in the click sample but absent in the nonclick control, or the abundance of the protein in the click sample was at least twofold greater than that in the nonclick control.

### Plasmids construction

For preparation of antibodies against viral proteins, full-length of *ADRV-85L*, *RGV-27R*, and *ADRV-23L* and a partial sequence of *ADRV-88L* were cloned into prokaryotic expression vectors. Specifically, full-length ORFs of *ADRV-85L*, *RGV-27R*, and *ADRV-23L* were amplified by PCR with FastPfu DNA polymerase (TransGen, China), and then enzyme digested and ligated into the pET32a vector. Partial sequences of *ADRV-88L* were amplified similarly but ligated into the pMAL-c5x vector.

To construct plasmids used in the NanoLuc complementation assay, the coding sequences of the N-terminus of NanoLuc (NlucN), polycloning sites (P, containing recognition sequences of EcoR I and BamH I), and Flag tag (F) and the coding sequences of the C-terminus of NanoLuc (NlucC), P, and HA tag (H) [38, 65] were codon-optimized based on the codon preference of zebra fish and synthesized (Sangon Biotech, China). The DNA sequences of NlucN, P, and F were amplified by PCR and ligated into the pcDNA3.1 vector to obtain two recombinant vectors, pcDNA3.1-NlucN-P-F and pcDNA3.1-P-NlucN-F. One vector contained an N-terminal NlucN in front of the cloning sites, and the other vector contained a C-terminal NlucN. The same method was used to obtain another two recombinant vectors, pcDNA3.1-NlucC-P-H and pcDNA3.1-P-NlucC-H. The coding sequences of viral and host genes were inserted into the obtained recombinant vectors to ensure that the genes had NlucN or NlucC in either orientation. The coding sequences of host genes were amplified by using reverse transcribed products from the extracted RNA of GSTC cells. An in-fusion cloning strategy was used in the construction of these plasmids.

For plasmids used in co-IP, full-length viral or host genes were cloned into the pcDNA3.1-3× Flag and pCGN-HAM vectors, respectively, to generate 3Flag- or 3HA-tagged proteins. All constructs were verified by DNA sequencing. The primers and resulting constructs are listed in Additional file 2: Table S1.

### Protein prokaryotic expression, purification, and antibody preparation

The recombinant plasmids for prokaryotic expression were transformed into *Escherichia coli* BL21 (DE3) and expressed, purified as previously described [66]. Positive clones were cultured in LB medium and induced with 0.1 mM isopropyl-β-d-thiogalactopyranoside (IPTG) for 8 h at 28 °C. The bacterial pellets were lysed by sonication. The recombinant proteins from the pET32a-based plasmids were purified using the HisBind Purification Kit (Novagen), and that from the pMAL-c5x-based plasmid was purified using the pMAL™ Protein Fusion and Purification System (NEB) according to the manufacturer’s instructions. Concentrations of purified proteins were determined using a BCA Protein Assay Kit (Beyotime, China) and stored at − 80 °C.

Recombinant RGV-27R protein was mixed with an equal volume of Freund’s adjuvant (Sigma) and used to immunize rabbits, while the recombinant ADRV-23L and ADRV-88L proteins were used to immunize mice with a standard immune procedure.

### Electrophoretic mobility shift assay (EMSA)

To detect the single-stranded DNA binding activity of ADRV-85L and RGV-27R, the two recombinant proteins were dialyzed in PBS and used in the EMSA, which was performed as described previously [67]. Each of the proteins was incubated with 1 μg of ΦX174 ssDNA (NEB) in a 20 μl reaction containing 12 mM Tris–Cl pH 8.0, 2.4% glycerol, 1 mM EDTA, and 2.5 mM β-mercaptoethanol. The mixtures were incubated for 20 min at 37 °C, and 2 μl of DNA loading buffer was added. The DNA–protein complexes were analyzed by 0.8% agarose gel electrophoresis. The DNA was stained with ethidium bromide and imaged.

### Western blotting

GSTC cells were infected with ADRV or RGV at an MOI of 0.1 and harvested at the indicated time points. The harvested cells were subjected to Western blotting as described previously [68]. The samples were analyzed by 12% SDS–PAGE and subsequently transferred to a PVDF membrane (Millipore). The polyclonal antibodies against viral proteins were used as the primary antibody, and the corresponding horseradish peroxidase (HRP)-conjugated goat anti-rabbit or anti-mouse IgG(H + L) (Merck) was used as the secondary antibody. Antibody binding was detected by chemiluminescence (Millipore). Determination of the expression of host Rpb3, Rpb6, and Rpb11 was performed as described above, and commercial antibody was used as the primary antibody.

### Immunofluorescence assay

Immunofluorescence assays were performed according to a previous study [69]. GSTC cells plated on coverslips were mock infected or infected with ADRV or RGV at an MOI of 0.5. The cells were fixed with 4% paraformaldehyde for 30 min at the indicated time points. Fixed cells were permeabilized with 0.5% Triton X-100 for 15 min, and then blocked in 10% normal goat serum for 1 h at room temperature. The cells were incubated with primary antibodies (anti-RGV-27R, anti-ADRV-23L, or anti-ADRV-88L) at a dilution of 1:100 in 2% normal goat serum for 2 h, rinsed three times for 10 min each with PBS containing 1% normal goat serum, and then incubated with secondary antibodies (Alexa-546-conjugated goat anti-rabbit IgG and Alexa-488-conjugated goat anti-mouse IgG) at a dilution of 1:1000. Hoechst 33342 was used to stain the nucleus. The final samples were examined under a Leica TCS SP8 confocal microscope.

### NanoLuc complementation assay

The NanoLuc complementation assay was performed in EPC and GSTC cells. Plasmids containing genes with NlucN and plasmids containing targeted genes with NlucC were cotransfected into cells in 24-well plates using Lipofectamine 3000 (Thermo Scientific, USA). The nanoluciferase activity was determined by using Nano-Glo^®^ Luciferase Assay Reagent (Promega, USA) according to the manufacturer’s instructions. Briefly, the cells were transferred to 96-well assay plates 48 h post transfection (hpt). Equal volumes of Nano-Glo^®^ Luciferase Assay Reagent were added to each well. Luminescence was recorded in a SpectraMax i3x Multi-Mode Microplate Reader (MD, USA). Cells cotransfected with vector and targeted genes were used as controls. All complementation assays were performed in triplicate. Data were presented as means ± SD. P values were calculated by using Student’s t test.

### Coimmunoprecipitation (co-IP) assay

Co-IP assays were performed in HEK293T cells as described in a previous study [27]. Briefly, HEK293T cells seeded in 6-well plates were cotransfected with 1.25 μg of 3Flag-tagged plasmid and 1.25 μg of 3HA-tagged plasmid. The corresponding empty vector was cotransfected in parallel as a control. At 24 hpt, cells were collected and lysed with a radioimmunoprecipitation assay (RIPA) buffer (Beyotime) containing PMSF and protease inhibitor cocktail (Sigma). The cell lysates were centrifuged at 16,000×*g* for 5 min at 4 °C, and the supernatants were incubated with red anti-HA affinity gel or anti-FLAG M2 affinity gel (Sigma) overnight at 4 °C. The precipitates were collected, washed with ice-cold PBST buffer five times, mixed with 30 μl of PBS, and subjected to Western blot analysis as described above. Commercial anti-HA (Abcam) or anti-Flag antibody (Sigma) was used as the primary antibody.

### Drug inhibition assay

The topoisomerase II inhibitor doxorubicin (MedChemExpress) was used as previously described [70]. Cytotoxicity was evaluated in GSTC cells by the CCK-8 assay [71]. Confluent cells in 96-well plates were treated with increasing concentrations of doxorubicin ranging from 0 to 300 nM for 24 h. After incubation, CCK-8 solution (Dojindo, Japan) was added and incubated for another 4 h. Colorimetric measurements were performed on a microplate reader at 450 nm. The cell viability was calculated for each concentration as OD_T_/OD_0_, where OD_T_ and OD_0_ correspond to the absorbance of drug-treated and non-drug-treated cells, respectively.

For the drug inhibition assay, confluent GSTC cells in 24-well plates were treated with different concentrations of doxorubicin for 1 h, followed by infection with recombinant ADRV expressing the mCherry tag at its TK gene locus (no published data) at 0.5 MOI. The insertion of exogenous genes in the TK locus of ranavirus has been proven to have no effect on virus replication in vitro*.* The infected cells were incubated for another 24 h with the drug. After incubation, the cells were observed under an Olympus IX73 fluorescence microscope and collected for virus titer and genome copy number determination.

The virus titers were determined using a 50% tissue culture infectious dose (TCID_50_) assay as described previously [63]. Total DNA was extracted with the TakaRa MiniBEST Universal Genomic DNA Extraction Kit (TakaRa, Japan). Viral genomes, based on detection of the relative numbers of major capsid protein gene (MCP), were detected by qPCR conducted using a StepOne Real-Time PCR system (Applied Biosystems, USA) as described previously [68]. The β-actin gene was used as a loading control, as in a previous study [72]. MCP levels were normalized to β-actin levels in each sample. The level of the viral genome (MCP level) in the treated group versus that in the control group (no drug) was calculated by the 2^−ΔΔCT^ method [73]. All experiments here and above were performed in triplicate. Data were presented as means ± SD. P values were calculated by using Student’s t test.

### Generation of recombinant ADRV with 46R-3Flag

The DNA sequences of the genome region before the initiation codon of *ADRV 46R* (46R_-L_) and after the stop codon (46R_-R_) and *P50-GFP* [74] were amplified separately by PCR. The 3xFlag coding sequence was synthesized within the reverse primer and added to 46R_-L_ during PCR amplification. These fragments were ligated into the pMD18-T vector by an infusion cloning strategy to obtain the recombinant plasmid pMD18T-46R_-L_-3xFlag-GFP-P50-46R_-R_, which was verified by DNA sequencing.

GSTC cells were transfected with the plasmid and then infected with ADRV at an MOI of 1 at 6 h post transfection. Cells were harvested at 48 hpi and diluted to infect GSTC cells in 24-well plates. Infected cells were covered with 0.75% melted soft agar and observed under a fluorescence microscope at 3 days post infection. The plaques emitting green fluorescence were selected to infect fresh GSTC cells and were purified as described above. The recombinant virus ADRV_46R-3Flag_ was purified by five rounds of plaque isolation. Expression of the 46R-3Flag protein was detected by Western blotting.

### Identification of 46R-3Flag associated proteins

To purify 46R-3Flag associated proteins, 2 × 10^8^ GSTC cells were infected with ADRV_46R-3Flag_ or wild type ADRV at an MOI of 1. Cells were collected at 24 hpi and resuspended in lysis buffer (50 mM HEPES, pH 7.5, 150 mM NaCl, 1.5 mM MgCl_2_, 0.5% NP-40, and 1 mM DTT) with EDTA-free protease inhibitor cocktail (Promega, USA). The cell lysates were centrifuged at 12,000×*g* for 15 min at 4 °C, and the supernatants were incubated with anti-FLAG M2 affinity gel (Sigma, USA) for 3 h at 4 °C. Then, the gel was washed four times with wash buffer (50 mM HEPES, pH 7.5, 150 mM NaCl, 1.5 mM MgCl_2_, 0.1% NP-40, and 1 mM DTT) and equilibrated with elution buffer (50 mM HEPES, pH 7.5, 150 mM NaCl, 1.5 mM MgCl_2_, and 1 mM DTT). The bead-bound proteins were eluted with 3× Flag peptide (Sigma, USA). The eluted proteins were analyzed with MS as described above. The assays were performed in triplicate.

### RNA interference

RNAi was performed in BHK-21 cells. First, siRNAs targeting Rpb3 (XM_040740763.1), Rpb6 (XM_005066978.4), and Rpb11 (XM_005080381.4) of *Mesocricetus auratus* were designed and synthesized (GenePharma, China). Sequences of the siRNAs were collected in Additional file 3: Table S2. The siRNAs were transfected into BHK-21 cells at a concentration of 25 nM using Lipofectamine RNAiMAX (Thermo Fisher, USA). Then, the cells were collected at 30 hpt to perform Western blot analysis. Alternatively, the cells were infected with ADRV (0.1 MOI) and collected at indicated time points. RT–qPCR was performed as described above. The primers are listed in Additional file 2: Table S1.

## Supplementary Information

**Additional file 1: Fig. S1.** Visualization of DNA labeled by EdU in RGV infections at 6 and 12 hpi. GSTC cells were infected with RGV at 1 MOI. The cells were incubated for 1 h with 10 μM EdU at the indicated time points and then fixed, permeabilized, and reacted with Alexa Fluor 488 azide. Cellular DNA was stained with Hoechst 33342 (blue). The EdU-labeled DNA is shown in green. The visible Hoechst-labeled cytoplasmic viral factories are indicated with arrows. **Fig. S2.** EdU labeling of ADRV at different times post infection. ADRV-infected GSTC cells were labeled with EdU at the indicated time points for 1 h. EdU-labeled nascent DNA is shown in green. Hoechst 33342-stained DNA is shown in blue. Normal GSTC cells were used as a control under the same processing. With lasting infection, the number of EdU-labeled nuclei decreased, while the number, size, and intensity of the cytoplasmic foci increased. The green color was located completely in the cytoplasm at 6–7 hpi. **Fig. S3.** EdU labeling of ADRV-infected cells at different treatment times. Infected GSTC cells were labeled with EdU for different periods (10 min, 20 min, and 30 min) at 12 hpi. The nuclei were stained with Hoechst 33342 (blue). EdU-labeled nascent DNA is presented in green. The 30 min labeling resulted in strong signals. **Fig. S4.** Characterization of RGV-27R. A. Prokaryotic expression and purification of recombinant RGV-27R. The protein markers, bacteria without induction (Uninduced), bacteria with induction (Induced), and purified proteins (Purified) are labeled at the top. The recombinant proteins with molecular weights of approximately 50 kDa are indicated with asterisks. B. Electrophoretic mobility shift analysis of the DNA–protein complexes. The ΦX174 DNA–protein complexes migrated more slowly with increasing protein amounts (0‒10 μg). C. Temporal expression of RGV-27R in virus infected GSTC cells by Western blot analysis. D. Subcellular localization of RGV-27R in virus infected GSTC cells by immunofluorescence. Viral nascent DNA was labeled with EdU as described above (green). RGV-27R was detected with anti-vSSB antibody (red). Cell nuclei were stained with Hoechst 33342 (blue). The visible Hoechst-labeled cytoplasmic viral factories are indicated with arrows. **Fig. S5.** Domain organization of core replisome components ADRV-88L/RGV-24R and ADRV-47L/RGV-63R. The domains (gray box) were searched using MOTIF Search in the Pfam and PROSITE databases. ADRV-88L/RGV-24R has a length of 975 aa and contains Primase_C, D5_N, and SF3 helicase domains. ADRV-47L/RGV-63R has a length of 1013 aa and contains exonuclease and DNA polymerase domains. Numbers at the top indicate the amino acid sites of the searched domains. **Fig. S6.** Temporal expression and localization of RGV-24R and RGV-91R. A. Western blot analysis of vHelicase/primase (RGV-24R) and vPCNA (RGV-91R). GSTC cells were infected by RGV at 0.5 MOI and collected at indicated time points. B. Localization of RGV-24R by immunofluorescence in GSTC cells. RGV-24R (green) colocalized with the vSSB (red). Representative overlaps are indicated by white arrows. C. Localization of RGV-91R by immunofluorescence. RGV-91R (green) colocalized with the vSSB (red). Representative overlaps are indicated by white arrows. The visible Hoechst-labeled cytoplasmic viral factories are indicated with yellow arrows. **Fig. S7.** Localization of Rpb3, Rpb6, and Rpb11 in RGV infected BHK-21 cells. The cells were infected with RGV at an MOI of 0.5 and fixed at the indicated time points. Rpb3, Rpb6, and Rpb11 (red) were stained with commercial antibodies. Replicating DNA was stained with EdU (green) as described above. Cell nuclei were stained with DAPI (blue). Bar = 5 μM. Rpb3, Rpb6, and Rpb11 were located in viral factories during RGV infection. **Fig. S8.** Gel electrophoresis of the deproteinized DNA. In the iPOND assay, cross-links were reversed in lysates collected after DNA sonication. The bound proteins were digested, and the DNA fragments were separated on a 1% agarose gel. The main molecular weights of the DNA fragments in the present study are from 250 to 500 bp.

**Additional file 2: Table S1.** Primers and constructs used in the study.

**Additional file 3: Table S2.** Sequences of the siRNAs used in the study.

## Data Availability

MS raw data files and result files have been deposited into iProX (https://iprox.org/) with dataset ID IPX0002798000 and into the ProteomeXchange Consortium (http://proteomecentral.proteomexchange.org) via the iProX partner repository [75] with the dataset identifier PXD024049.

## Abbreviations

ADRV: *Andrias davidianus* Ranavirus
RGV: *Rana grylio* Virus
FV3: Frog virus 3
NCLDVs: Nucleo-cytoplasmic large DNA viruses
iPOND: Isolation of proteins on nascent DNA
Nluc: NanoLuciferase
SSB: Single-stranded binding protein
RNAP II: DNA-dependent RNA polymerase II
PCNA: Proliferating cell nuclear antigen
TopIIα: Topoisomerase IIα
TopIIβ: Topoisomerase IIβ
GSTC: Chinese giant salamander thymus cell line
BHK-21: Baby hamster kidney fibroblast cell line
RNAi: RNA interference
EPC: *Epithelioma papulosum cyprini* cell line
HEK293T: Human embryonic kidney cell line

## References

[1] Chinchar VG, Waltzek TB (2014). Ranaviruses: not just for frogs. PLoS Pathog.

[2] Marschang RE, Becher P, Posthaus H, Wild P, Thiel HJ, Müller-Doblies U, Kalet EF, Bacciarini LN (1999). Isolation and characterization of an iridovirus from Hermann's tortoises (*Testudo hermanni*). Arch Virol.

[3] Huang Y, Huang X, Liu H, Gong J, Ouyang Z, Cui H, Cao J, Zhao Y, Wang X, Jiang Y (2009). Complete sequence determination of a novel reptile iridovirus isolated from soft-shelled turtle and evolutionary analysis of *Iridoviridae*. BMC Genomics.

[4] Zhang QY, Xiao F, Li ZQ, Gui JF, Mao J, Chinchar VG (2001). Characterization of an iridovirus from the cultured pig frog *Rana grylio* with lethal syndrome. Dis Aquat Organ.

[5] Chinchar VG, Yu KH, Jancovich JK (2011). The molecular biology of frog virus 3 and other iridoviruses infecting cold-blooded vertebrates. Viruses.

[6] Chen ZY, Gui JF, Gao XC, Pei C, Hong YJ, Zhang QY (2013). Genome architecture changes and major gene variations of *Andrias davidianus* ranavirus (ADRV). Vet Res.

[7] Chinchar VG, Hick P, Ince IA, Jancovich JK, Marschang R, Qin Q, Subramaniam K, Waltzek TB, Whittington R, Williams T (2017). ICTV virus taxonomy profile: *Iridoviridae*. J Gen Virol.

[8] George MR, John KR, Mansoor MM, Saravanakumar R, Sundar P, Pradeep V (2015). Isolation and characterization of a ranavirus from koi, *Cyprinus carpio* L., experiencing mass mortalities in India. J Fish Dis.

[9] Qin QW, Chang SF, Ngoh-Lim GH, Gibson-Kueh S, Shi C, Lam TJ (2003). Characterization of a novel ranavirus isolated from grouper *Epinephelus tauvina*. Dis Aquat Org.

[10] Deng L, Geng Y, Zhao R, Gray MJ, Wang K, Ouyang P, Chen D, Huang X, Chen Z, Huang C (2020). CMTV-like ranavirus infection associated with high mortality in captive catfish-like loach, *Triplophysa siluorides* in China. Transbound Emerg Dis.

[11] Zhang QY, Gui JF (2015). Virus genomes and virus-host interactions in aquaculture animals. Sci China Life Sci.

[12] Price SJ, Ariel E, Maclaine A, Rosa GM, Gray MJ, Brunner JL, Garner TWJ (2017). From fish to frogs and beyond: impact and host range of emergent ranaviruses. Virology.

[13] Schock DM, Bollinger TK, Gregory Chinchar V, Jancovich JK, Collins JP (2008). Experimental evidence that amphibian ranaviruses are multi-host pathogens. Copeia.

[14] Claytor SC, Subramaniam K, Landrau-Giovannetti N, Chinchar VG, Gray MJ, Miller DL, Mavian C, Salemi M, Wisely S, Waltzek TB (2017). Ranavirus phylogenomics: signatures of recombination and inversions among bullfrog ranaculture isolates. Virology.

[15] Carstairs SJ, Kyle CJ, Vilaca ST (2020). High prevalence of subclinical frog virus 3 infection in freshwater turtles of Ontario, Canada. Virology.

[16] Vilaca ST, Bienentreu JF, Brunetti CR, Lesbarreres D, Murray DL, Kyle CJ (2019). Frog virus 3 genomes reveal prevalent recombination between ranavirus lineages and their origins in Canada. J Virol.

[17] Koonin EV, Krupovic M, Ishino S, Ishino Y (2020). The replication machinery of LUCA: common origin of DNA replication and transcription. BMC Biol.

[18] van Oijen AM, Loparo JJ (2010). Single-molecule studies of the replisome. Annu Rev Biophys.

[19] Zhang D, O'Donnell M (2016). The eukaryotic replication machine. Enzymes.

[20] Kornberg RD (2007). The molecular basis of eukaryotic transcription. Proc Natl Acad Sci USA.

[21] Svetlov V, Nudler E (2013). Basic mechanism of transcription by RNA polymerase II. Bba-Gene Regul Mech.

[22] Dremel SE, DeLuca NA (2019). Genome replication affects transcription factor binding mediating the cascade of herpes simplex virus transcription. Proc Natl Acad Sci USA.

[23] Sun W, Huang YH, Zhao Z, Gui JF, Zhang QY (2006). Characterization of the *Rana grylio* virus 3beta-hydroxysteroid dehydrogenase and its novel role in suppressing virus-induced cytopathic effect. Biochem Biophys Res Commun.

[24] Zhao Z, Ke F, Gui J, Zhang Q (2007). Characterization of an early gene encoding for dUTPase in *Rana grylio* virus. Virus Res.

[25] Zhao Z, Ke F, Shi Y, Zhou GZ, Gui JF, Zhang QY (2009). Rana grylio virus thymidine kinase gene: an early gene of iridovirus encoding for a cytoplasmic protein. Virus Genes.

[26] Lei XY, Ou T, Zhang QY (2012). *Rana grylio* virus (RGV) 50L is associated with viral matrix and exhibited two distribution patterns. PLoS ONE.

[27] Zeng XT, Zhang QY (2019). Interaction between two iridovirus core proteins and their effects on ranavirus (RGV) replication in cells from different species. Viruses.

[28] Zhang R, Zhang QY (2018). Adenosine triphosphatase activity and cell growth promotion of *Andrias davidianus* ranavirus 96L-encoded protein (ADRV-96L). Microbiol China.

[29] Whitley DS, Sample RC, Sinning AR, Henegar J, Chinchar VG (2011). Antisense approaches for elucidating ranavirus gene function in an infected fish cell line. Dev Comp Immunol.

[30] Sirbu BM, Couch FB, Cortez D (2012). Monitoring the spatiotemporal dynamics of proteins at replication forks and in assembled chromatin using isolation of proteins on nascent DNA. Nat Protoc.

[31] Dembowski JA, DeLuca NA (2015). Selective recruitment of nuclear factors to productively replicating herpes simplex virus genomes. PLoS Pathog.

[32] Dembowski JA, DeLuca NA (2018). Temporal viral genome-protein interactions define distinct stages of productive herpesviral infection. MBio.

[33] Reyes ED, Kulej K, Pancholi NJ, Akhtar LN, Avgousti DC, Kim ET, Bricker DK, Spruce LA, Koniski SA, Seeholzer SH (2017). Identifying host factors associated with DNA replicated during virus infection. Mol Cell Proteomics.

[34] Senkevich TG, Katsafanas GC, Weisberg A, Olano LR, Moss B (2017). Identification of vaccinia virus replisome and transcriptome proteins by isolation of proteins on nascent DNA coupled with mass spectrometry. J Virol.

[35] Xu HZ, Perez RD, Frey TR, Burton EM, Mannemuddhu S, Haley JD, McIntosh MT, Bhaduri-McIntosh S (2019). Novel replisome-associated proteins at cellular replication forks in EBV-transformed B lymphocytes. PLoS Pathog.

[36] Huang XH, Huang YH, Yuan XP, Zhang QY (2006). Electron microscopic examination of the viromatrix of Rana grylio virus in a fish cell line. J Virol Methods.

[37] Tan WG, Barkman TJ, Gregory Chinchar V, Essani K (2004). Comparative genomic analyses of frog virus 3, type species of the genus Ranavirus (family Iridoviridae). Virology.

[38] Dixon AS, Schwinn MK, Hall MP, Zimmerman K, Otto P, Lubben TH, Butler BL, Binkowski BF, Machleidt T, Kirkland TA (2016). NanoLuc complementation reporter optimized for accurate measurement of protein interactions in cells. ACS Chem Biol.

[39] Elliott RM, Bravo R, Kelly DC (1980). Frog virus 3 replication: analysis of structural and nonstructural polypeptides in infected BHK cells by acidic and basic two-dimensional gel electrophoresis. J Virol.

[40] Ke F, Gui JF, Chen ZY, Li T, Lei CK, Wang ZH, Zhang QY (2018). Divergent transcriptomic responses underlying the ranaviruses-amphibian interaction processes on interspecies infection of Chinese giant salamander. BMC Genomics.

[41] Goorha R (1982). Frog virus 3 DNA replication occurs in two stages. J Virol.

[42] Hutin S, Ling WL, Round A, Effantin G, Reich S, Iseni F, Tarbouriech N, Schoehn G, Burmeister WP (2016). Domain organization of vaccinia virus helicase-primase D5. J Virol.

[43] De Silva FS, Lewis W, Berglund P, Koonin EV, Moss B (2007). Poxvirus DNA primase. Proc Natl Acad Sci USA.

[44] Gonzalez-Magana A, Blanco FJ (2020). Human PCNA structure, function and interactions. Biomolecules.

[45] Dixon LK, Chapman DA, Netherton CL, Upton C (2013). African swine fever virus replication and genomics. Virus Res.

[46] Moss B (2013). Poxvirus DNA replication. Cold Spring Harb Perspect Biol.

[47] Stanitsa ES, Arps L, Traktman P (2006). Vaccinia virus uracil DNA glycosylase interacts with the A20 protein to form a heterodimeric processivity factor for the viral DNA polymerase. J Biol Chem.

[48] Rochester SC, Traktman P (1998). Characterization of the single-stranded DNA binding protein encoded by the vaccinia virus I3 gene. J Virol.

[49] Chu WT, Suo ZC, Wang J (2020). Binding-induced conformational changes involved in sliding clamp PCNA and DNA polymerase DPO4. Iscience.

[50] Kilkenny ML, De Piccoli G, Perera RL, Labib K, Pellegrini L (2012). A conserved motif in the C-terminal tail of DNA polymerase alpha tethers primase to the eukaryotic replisome. J Biol Chem.

[51] Cerron F, de Lorenzo S, Lemishko KM, Ciesielski GL, Kaguni LS, Cao FJ, Ibarra B (2019). Replicative DNA polymerases promote active displacement of SSB proteins during lagging strand synthesis. Nucleic Acids Res.

[52] Naue N, Beerbaum M, Bogutzki A, Schmieder P, Curth U (2013). The helicase-binding domain of *Escherichia coli* DnaG primase interacts with the highly conserved C-terminal region of single-stranded DNA-binding protein. Nucleic Acids Res.

[53] Schier AC, Taatjes DJ (2020). Structure and mechanism of the RNA polymerase II transcription machinery. Genes Dev.

[54] Mirzakhanyan Y, Gershon PD (2017). Multisubunit DNA-dependent RNA polymerases from vaccinia virus and other nucleocytoplasmic large-DNA viruses: impressions from the age of structure. Microbiol Mol Biol Rev.

[55] Lei XY, Ou T, Zhu RL, Zhang QY (2012). Sequencing and analysis of the complete genome of *Rana grylio* virus (RGV). Arch Virol.

[56] Kimura M, Ishihama A (2000). Involvement of multiple subunit-subunit contacts in the assembly of RNA polymerase II. Nucleic Acids Res.

[57] Acker J, de Graaff M, Cheynel I, Khazak V, Kedinger C, Vigneron M (1997). Interactions between the human RNA polymerase II subunits. J Biol Chem.

[58] Williams RS, Kunkel TA (2011). FEN nucleases: bind, bend, fray, cut. Cell.

[59] Riccio AA, Schellenberg MJ, Williams RS (2020). Molecular mechanisms of topoisomerase 2 DNA-protein crosslink resolution. Cell Mol Life Sci.

[60] Caglayan M (2019). Interplay between DNA polymerases and DNA ligases: influence on substrate channeling and the fidelity of DNA ligation. J Mol Biol.

[61] Lin YC, Li J, Irwin CR, Jenkins H, DeLange L, Evans DH (2008). Vaccinia virus DNA ligase recruits cellular topoisomerase II to sites of viral replication and assembly. J Virol.

[62] Kaur K, Rohozinski J, Goorha R (1995). Identification and characterization of the frog virus-3 DNA methyltransferase gene. J Gen Virol.

[63] Zeng XT, Gao XC, Zhang QY (2018). Rana grylio virus 43R encodes an envelope protein involved in virus entry. Virus Genes.

[64] Yang MK, Yang YH, Chen Z, Zhang J, Lin Y, Wang Y, Xiong Q, Li T, Ge F, Bryant DA (2014). Proteogenomic analysis and global discovery of posttranslational modifications in prokaryotes. Proc Natl Acad Sci USA.

[65] Wang FZ, Zhang N, Guo YJ, Gong BQ, Li JF (2020). Split Nano luciferase complementation for probing protein-protein interactions in plant cells. J Integr Plant Biol.

[66] Ming CY, Ke F, Zhang QY (2019). The expression and immunogenic analysis of ranaviruses homologous proteins RGV-27R and ADRV-85L. Chin J Virol.

[67] Harrison ML, Desaulniers MA, Noyce RS, Evans DH (2016). The acidic C-terminus of vaccinia virus I3 single-strand binding protein promotes proper assembly of DNA-protein complexes. Virology.

[68] Ke F, Wang ZH, Ming CY, Zhang QY (2019). Ranaviruses bind cells from different species through interaction with heparan sulfate. Viruses.

[69] Ke F, He LB, Zhang QY (2013). Nonstructural protein NS80 is crucial in recruiting viral components to form aquareoviral factories. PLoS ONE.

[70] Sheraz M, Cheng JJ, Tang LD, Chang JH, Guo JT (2019). Cellular DNA topoisomerases are required for the synthesis of hepatitis B virus covalently closed circular DNA. J Virol.

[71] Ouyang P, Yang RX, Yin LZ, Geng YM, Lai WM, Huang XL, Chen DF, Fang J, Chen ZL, Tang L (2019). Molecular characterization of Cyprinid herpesvirus 3 encoded viral interleukin10. Fish Shellfish Immunol.

[72] Zhu R, Chen ZY, Wang J, Yuan JD, Liao XY, Gui JF, Zhang QY (2014). Extensive diversification of MHC in Chinese giant salamanders *Andrias davidianus* (Anda-MHC) reveals novel splice variants. Dev Comp Immunol.

[73] Livak KJ, Schmittgen TD (2001). Analysis of relative gene expression data using real-time quantitative PCR and the 2^−^^ΔΔ^^CT^ method. Methods.

[74] He LB, Ke F, Wang J, Gao XC, Zhang QY (2014). *Rana grylio* virus (RGV) envelope protein 2L: subcellular localization and essential roles in virus infectivity revealed by conditional lethal mutant. J Gen Virol.

[75] Ma J, Chen T, Wu S, Yang C, Bai M, Shu K, Li K, Zhang G, Jin Z, He F (2019). iProX: an integrated proteome resource. Nucleic Acids Res.

