# Supplementary material for: Replication and transcription machinery for ranaviruses: components, correlation, and functional architecture
Source: Cell Biosci. 2022 Jan 6;12:6. doi: 10.1186/s13578-021-00742-x (PMC8734342; doi:10.1186/s13578-021-00742-x)
Supplement: Supplementary file 1 — Additional file 1: Fig. S1. Visualization of DNA labeled by EdU in RGV infections at 6 and 12 hpi. GSTC cells were infected with RGV at 1 MOI. The cells were incubated for 1 h with 10 μM EdU at the indicated time points and then fixed, permeabilized, and reacted with Alexa Fluor 488 azide. Cellular DNA was stained with Hoechst 33342 (blue). The EdU-labeled DNA is shown in green. The visible Hoechst-labeled cytoplasmic viral factories are indicated with arrows. Fig. S2. EdU labeling of ADRV at different times post infection. ADRV-infected GSTC cells were labeled with EdU at the indicated time points for 1 h. EdU-labeled nascent DNA is shown in green. Hoechst 33342-stained DNA is shown in blue. Normal GSTC cells were used as a control under the same processing. With lasting infection, the number of EdU-labeled nuclei decreased, while the number, size, and intensity of the cytoplasmic foci increased. The green color was located completely in the cytoplasm at 6–7 hpi. Fig. S3. EdU labeling of ADRV-infected cells at different treatment times. Infected GSTC cells were labeled with EdU for different periods (10 min, 20 min, and 30 min) at 12 hpi. The nuclei were stained with Hoechst 33342 (blue). EdU-labeled nascent DNA is presented in green. The 30 min labeling resulted in strong signals. Fig. S4. Characterization of RGV-27R. A. Prokaryotic expression and purification of recombinant RGV-27R. The protein markers, bacteria without induction (Uninduced), bacteria with induction (Induced), and purified proteins (Purified) are labeled at the top. The recombinant proteins with molecular weights of approximately 50 kDa are indicated with asterisks. B. Electrophoretic mobility shift analysis of the DNA–protein complexes. The ΦX174 DNA–protein complexes migrated more slowly with increasing protein amounts (0‒10 μg). C. Temporal expression of RGV-27R in virus infected GSTC cells by Western blot analysis. D. Subcellular localization of RGV-27R in virus infected GSTC cells by immunof [file 13578_2021_742_MOESM1_ESM.docx]

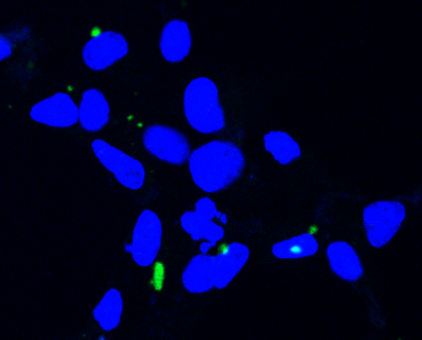

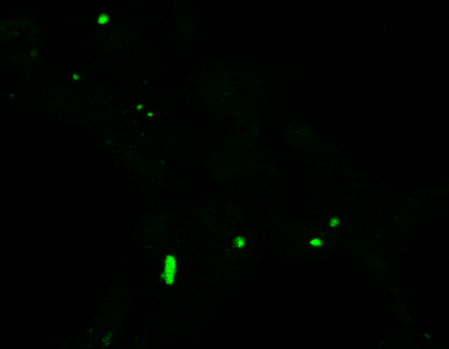

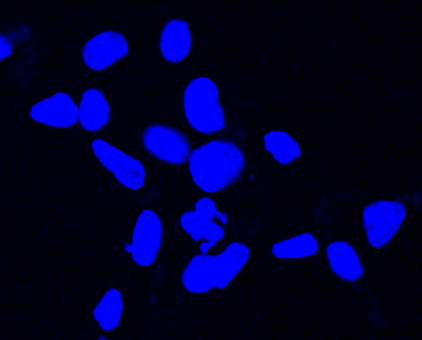

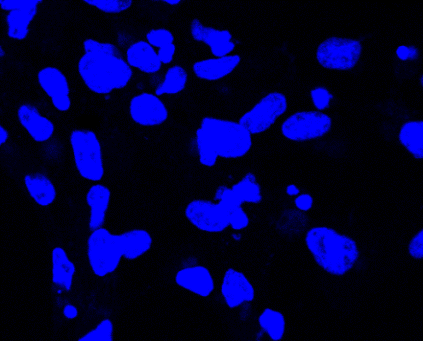

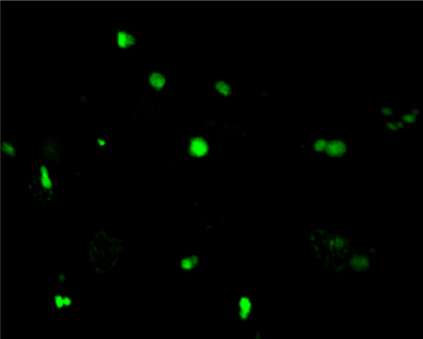

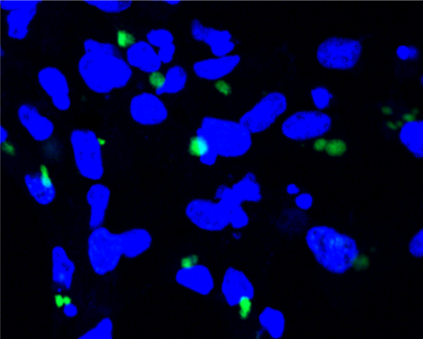


10 μm

**6 hpi**

**12 hpi**

**RGV**

**Fig. S1 Visualization of DNA labeled by EdU in RGV infections at 6 and 12 hpi.** GSTC cells were infected with RGV at 1 MOI. The cells were incubated for 1 h with 10 μM EdU at the indicated time points and then fixed, permeabilized, and reacted with Alexa Fluor 488 azide. Cellular DNA was stained with Hoechst 33342 (blue). The EdU-labeled DNA is shown in green. The visible Hoechst-labeled cytoplasmic viral factories are indicated with arrows.


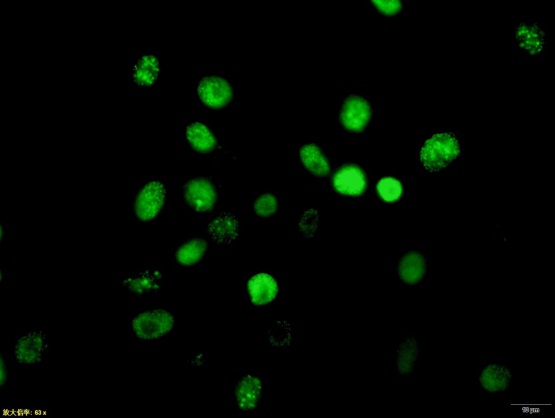

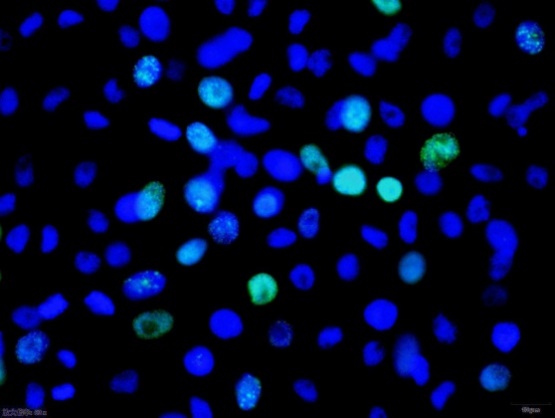

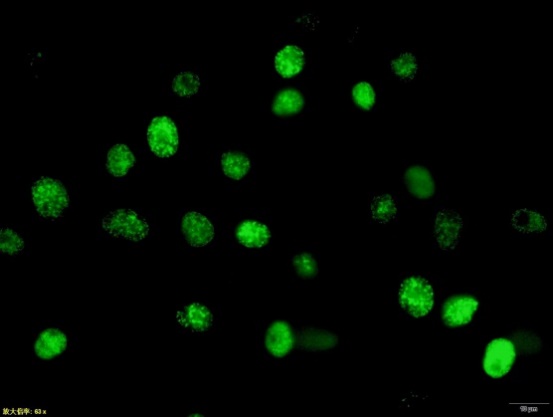

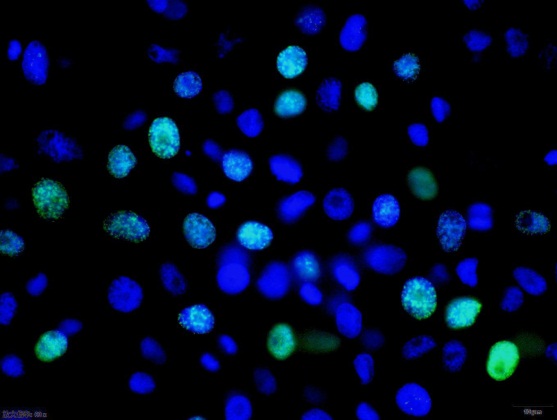

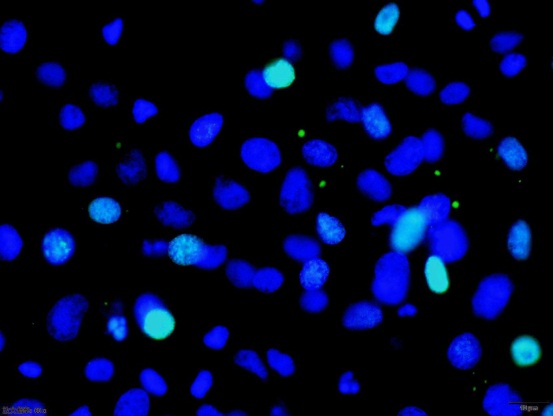

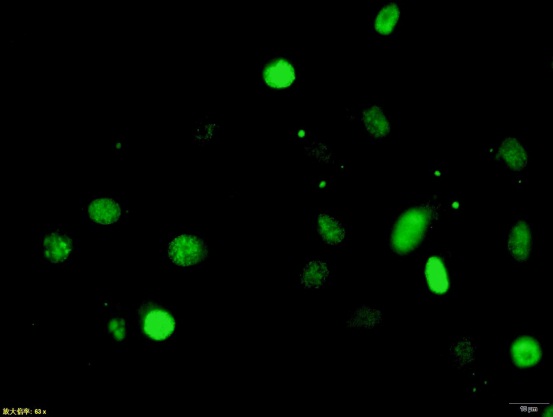

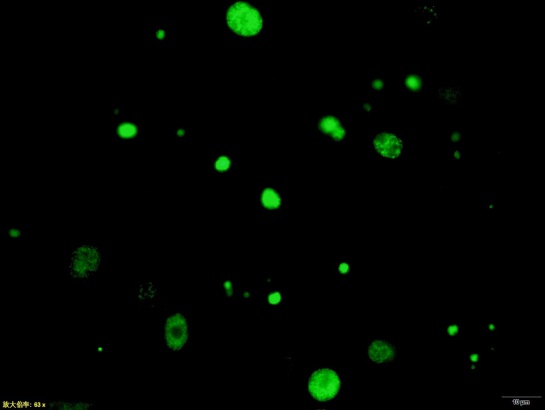

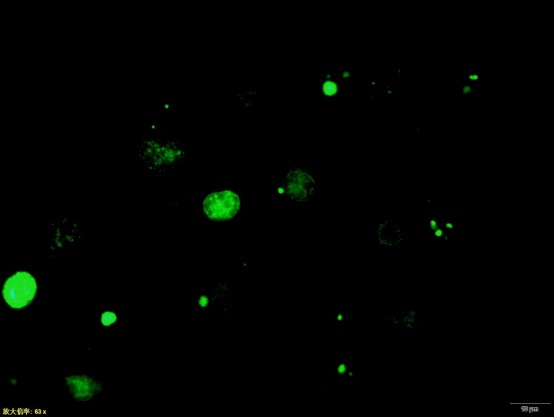

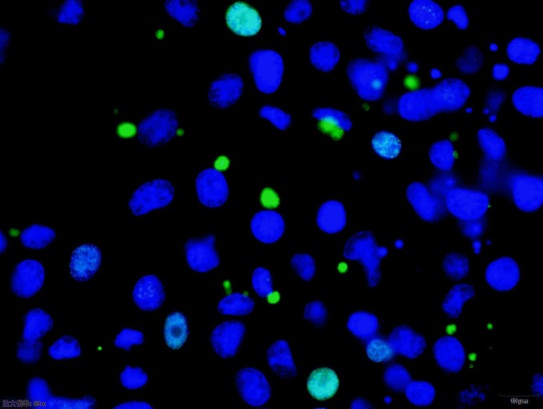

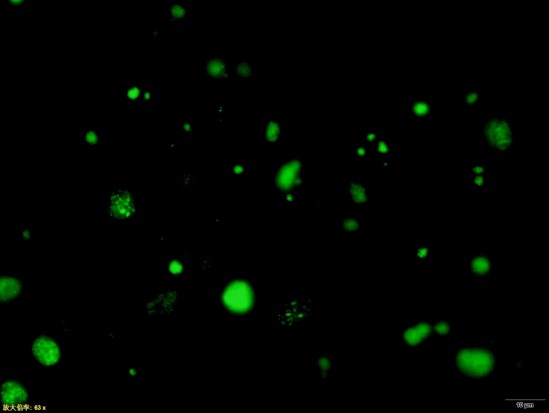


**control**

**1-2 hpi**

**5-6 hpi**


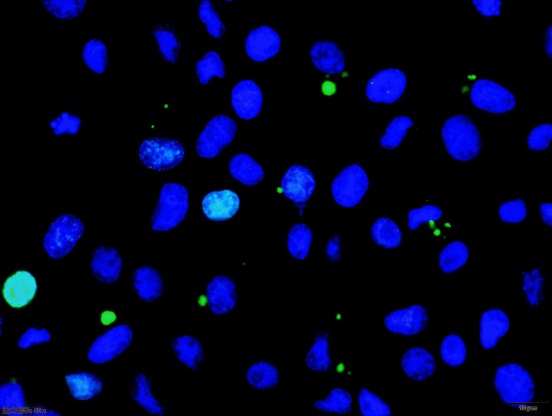


**3-4 hpi**


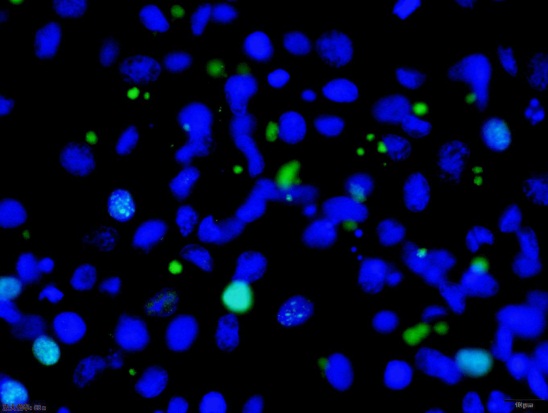


**4-5 hpi**

**6-7 hpi**

**Fig. S2 EdU labeling of ADRV at different times post infection.** ADRV-infected GSTC cells were labeled with EdU at the indicated time points for 1 h. EdU-labeled nascent DNA is shown in green. Hoechst 33342-stained DNA is shown in blue. Normal GSTC cells were used as a control under the same processing. With lasting infection, the number of EdU-labeled nuclei decreased, while the number, size, and intensity of the cytoplasmic foci increased. The green color was located completely in the cytoplasm at 6-7 hpi.


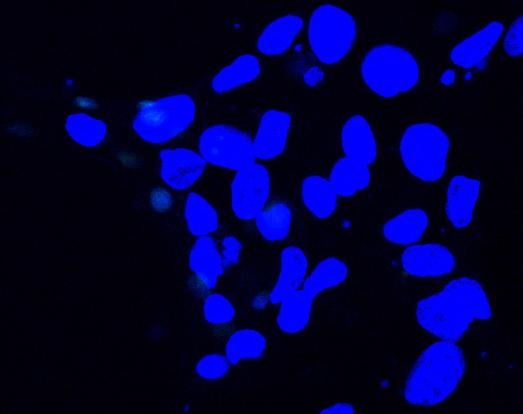

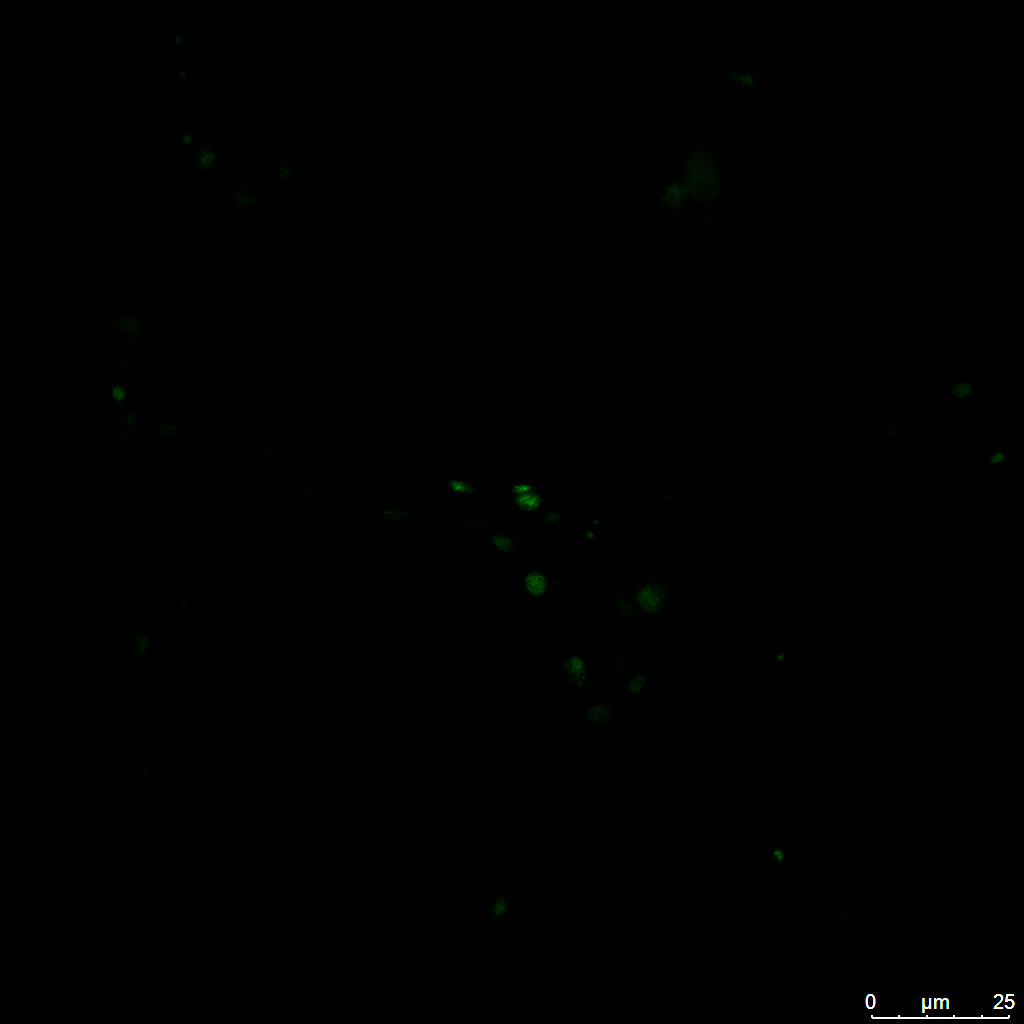

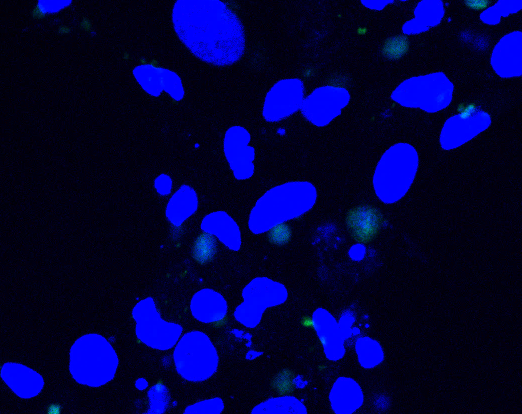

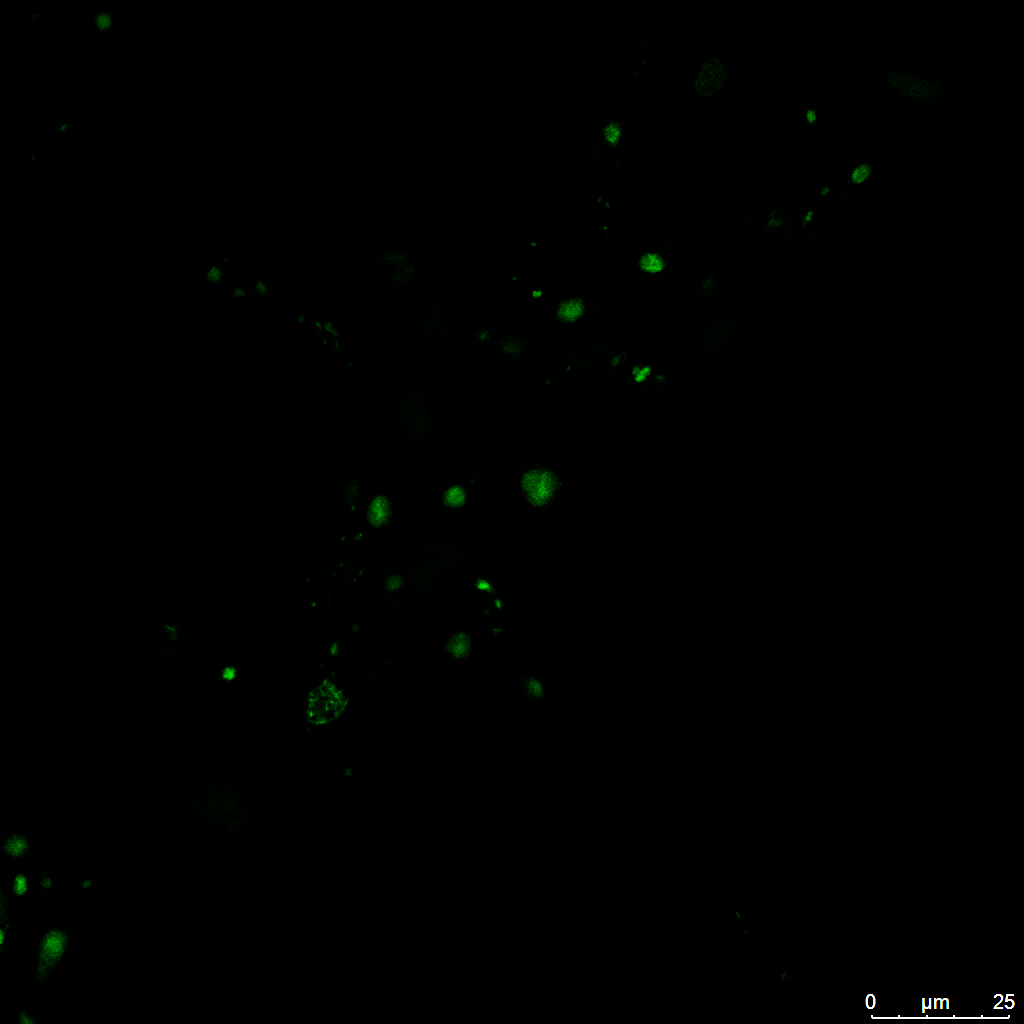

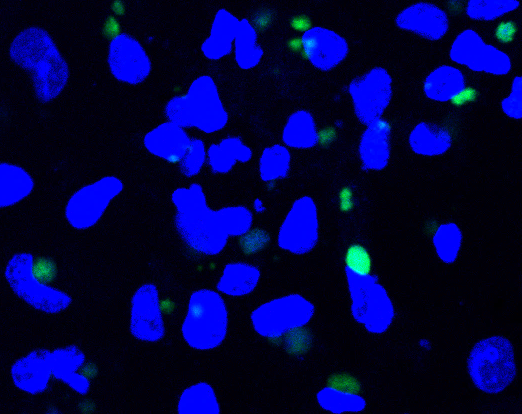

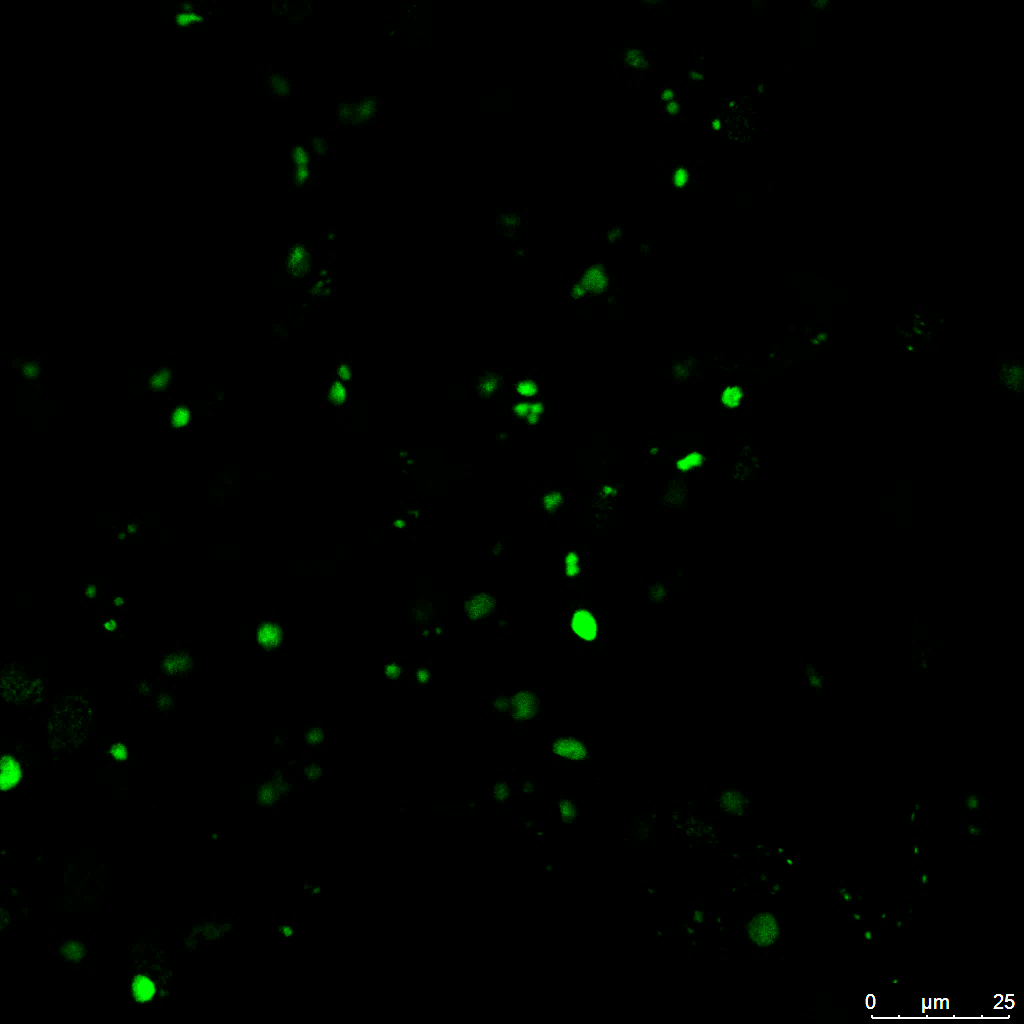


**10 min**

**20 min**

**30 min**

**Fig. S3 EdU labeling of ADRV-infected cells at different treatment times**. Infected GSTC cells were labeled with EdU for different periods (10 min, 20 min, and 30 min) at 12 hpi. The nuclei were stained with Hoechst 33342 (blue). EdU-labeled nascent DNA is presented in green. The 30 min labeling resulted in strong signals.

**RGV-27R**

M 0 1 2 4 6 8 10 μg


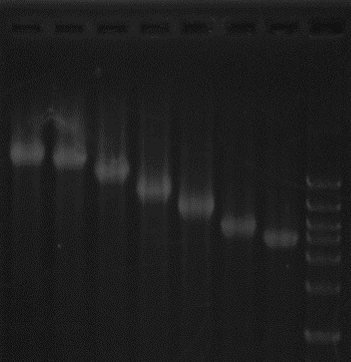


B


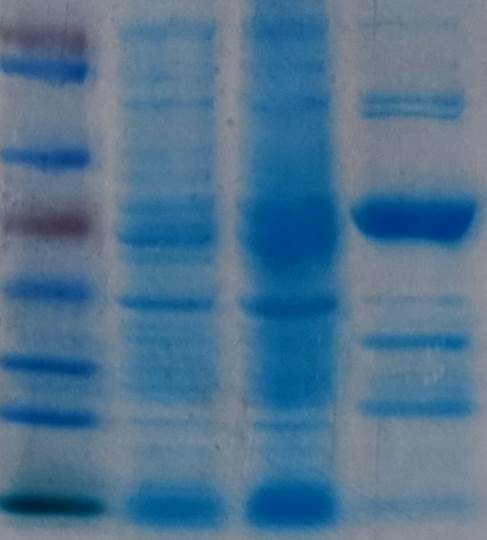


*

**RGV-27R**

Uninduced

Marker

Induced

Purified

A

C 0 2 4 6 8 12 24 48h


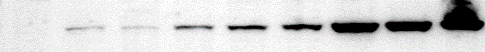


RGV-27R

β-actin


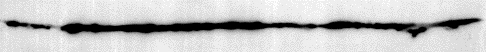


C


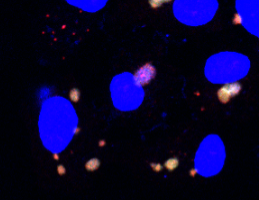

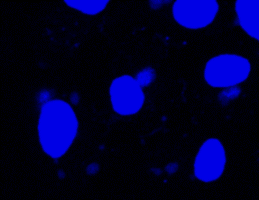

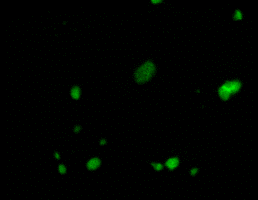

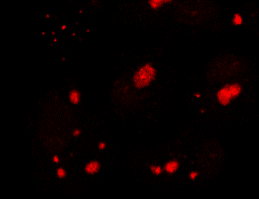


**EdU**

**RGV-27R**

**Hoechst**

**Merge**

D

10 μm


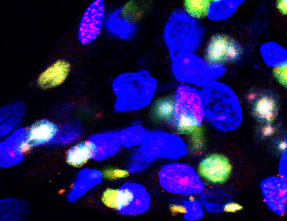

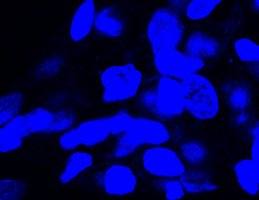

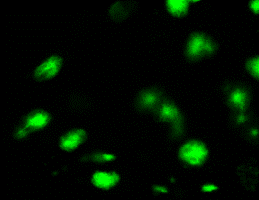

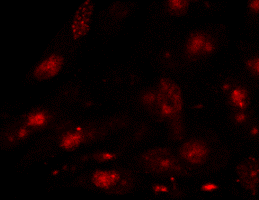


10 μm

**12 hpi**

**24 hpi**

**RGV**


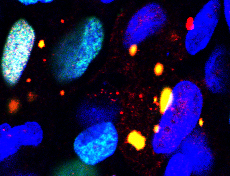

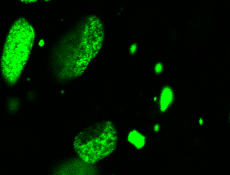

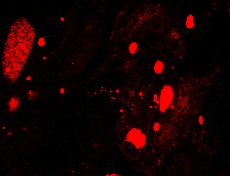

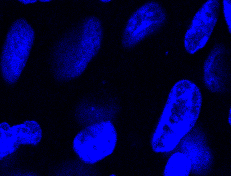


**4 hpi**


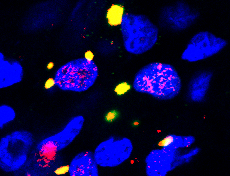

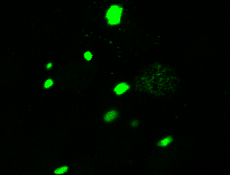

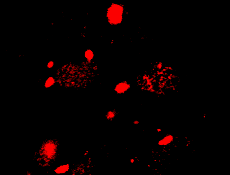

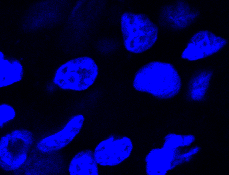


**6 hpi**

10 μm

10 μm

**Fig. S4 Characterization of RGV-27R**. A. Prokaryotic expression and purification of recombinant RGV-27R. The protein markers, bacteria without induction (Uninduced), bacteria with induction (Induced), and purified proteins (Purified) are labeled at the top. The recombinant proteins with molecular weights of approximately 50 kDa are indicated with asterisks. B. Electrophoretic mobility shift analysis of the DNA-protein complexes. The ΦX174 DNA-protein complexes migrated more slowly with increasing protein amounts (0‒10 μg). C. Temporal expression of RGV-27R in virus infected GSTC cells by Western blot analysis. D. Subcellular localization of RGV-27R in virus infected GSTC cells by immunofluorescence. Viral nascent DNA was labeled with EdU as described above (green). RGV-27R was detected with anti-vSSB antibody (red). Cell nuclei were stained with Hoechst 33342 (blue). The visible Hoechst-labeled cytoplasmic viral factories are indicated with arrows.

1

975

842

660

603

410

381

306

Primase_C

SF3 helicase

D5_N

ADRV-88L

RGV-24R

1

975

842

660

603

410

381

306

Primase_C

SF3 helicase

D5_N

1013

372

517

585

969

22

340

1

DNA Pol family B

exonuclease

ADRV-47L

RGV-63R

1013

372

511

586

969

22

340

1

DNA Pol family B

exonuclease

Fig. S5

**Fig. S5 Domain organization of core replisome components ADRV-88L/RGV-24R and ADRV-47L/RGV-63R.** The domains (gray box) were searched using MOTIF Search in the Pfam and PROSITE databases. ADRV-88L/RGV-24R has a length of 975 aa and contains Primase_C, D5_N, and SF3 helicase domains. ADRV-47L/RGV-63R has a length of 1013 aa and contains exonuclease and DNA polymerase domains. Numbers at the top indicate the amino acid sites of the searched domains.


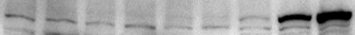


RGV-24R


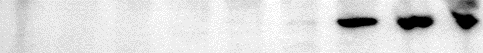


RGV-91R

C 0 2 4 6 8 12 24 48 h

A


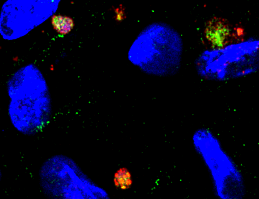

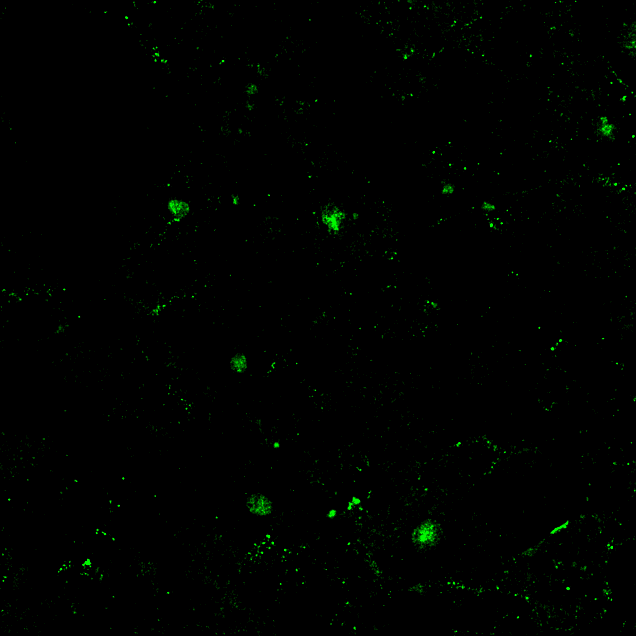

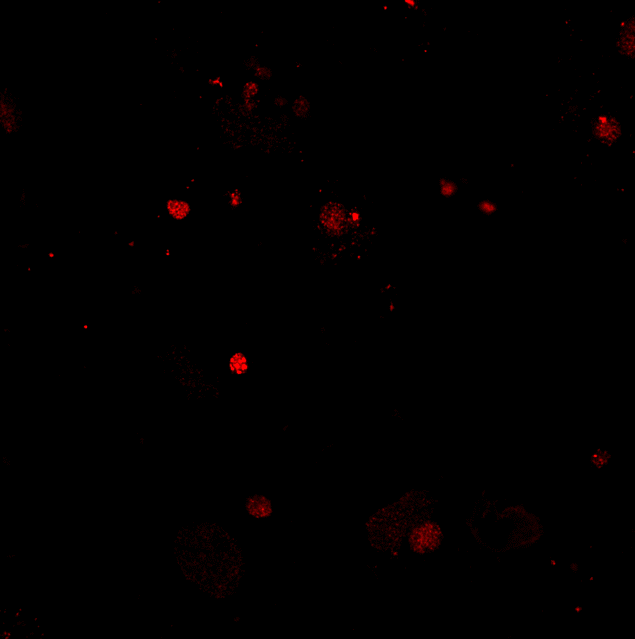

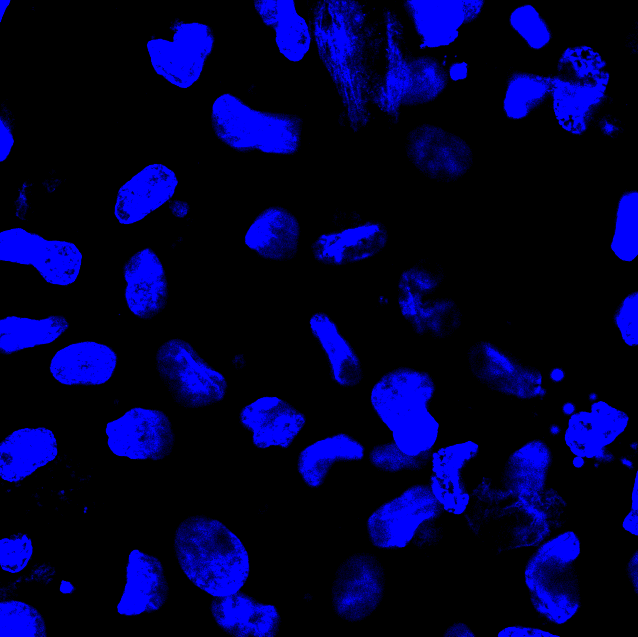

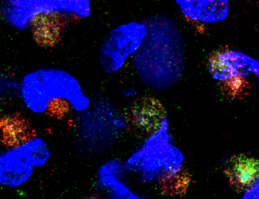

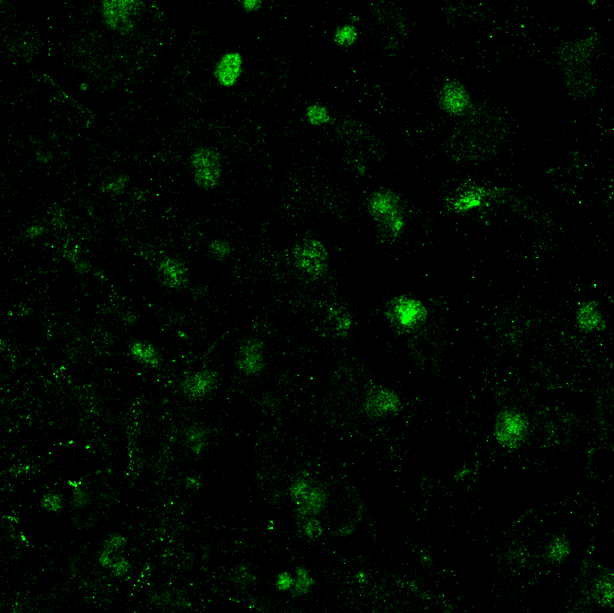

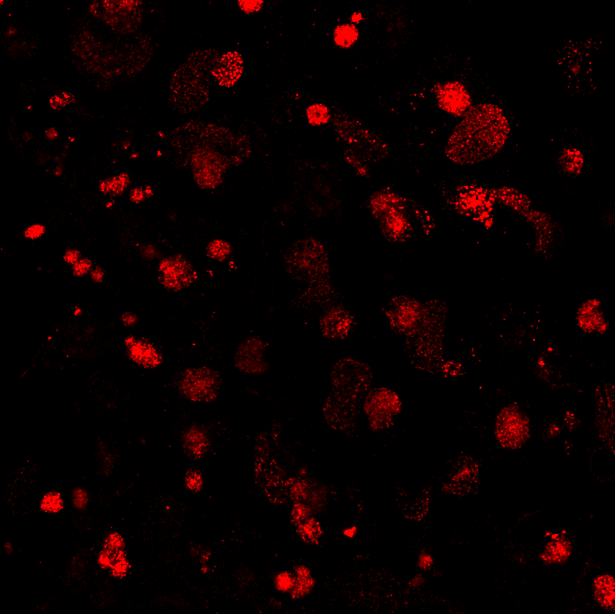

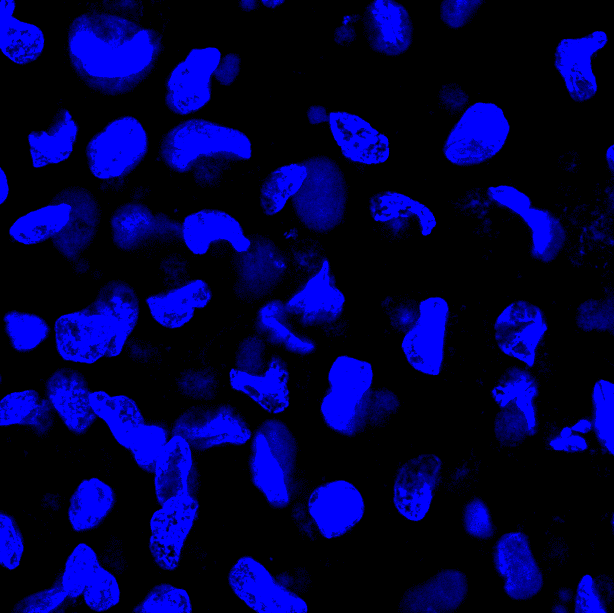


**RGV-24R**

**RGV-27R**

**Hoechst**

**Merge**

5 μm

5 μm

**12 hpi**

**24 hpi**

**RGV**

B


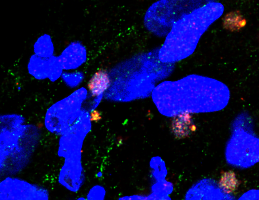

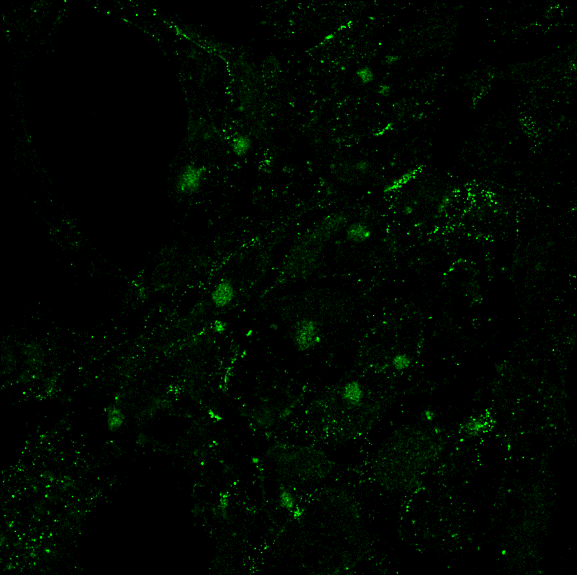

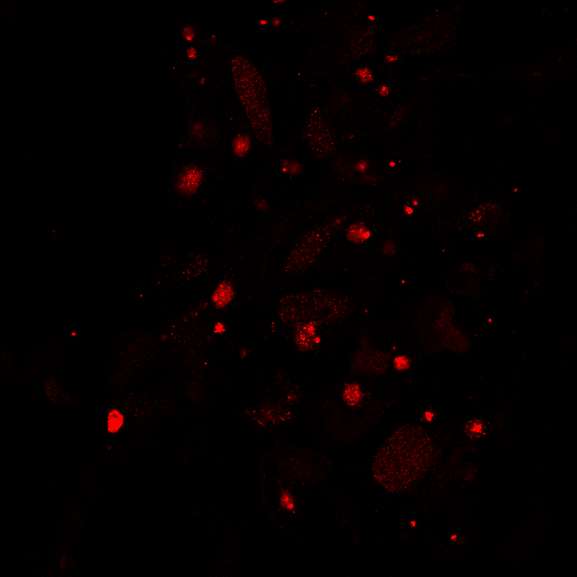

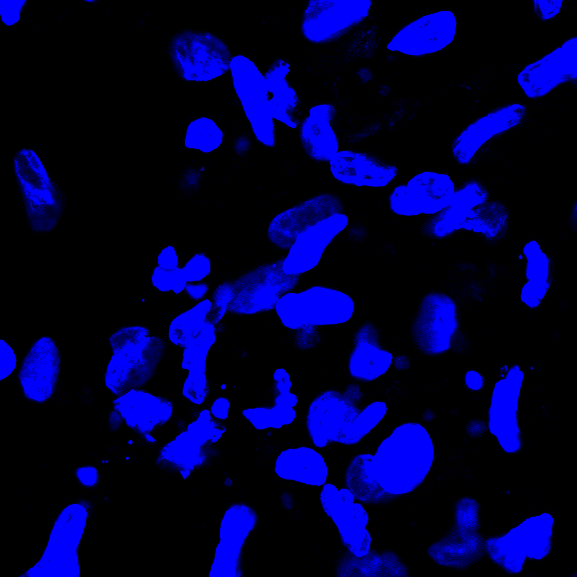

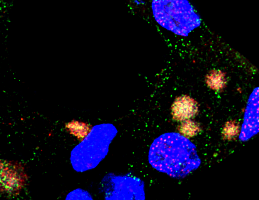

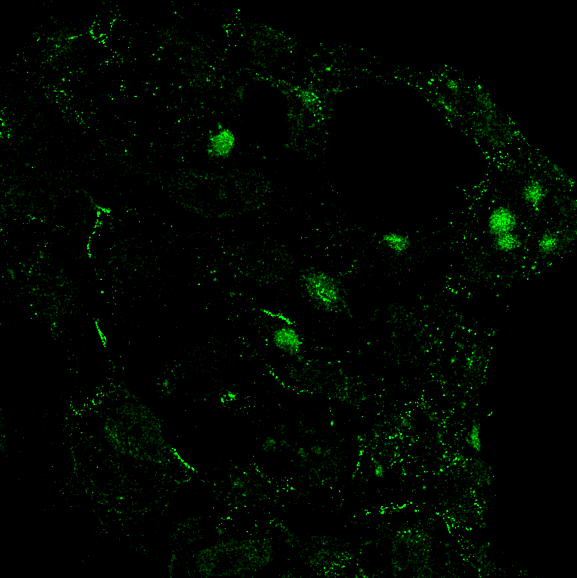

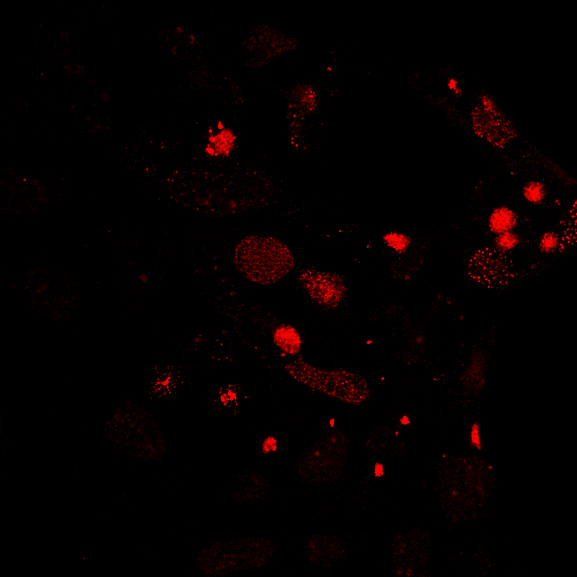

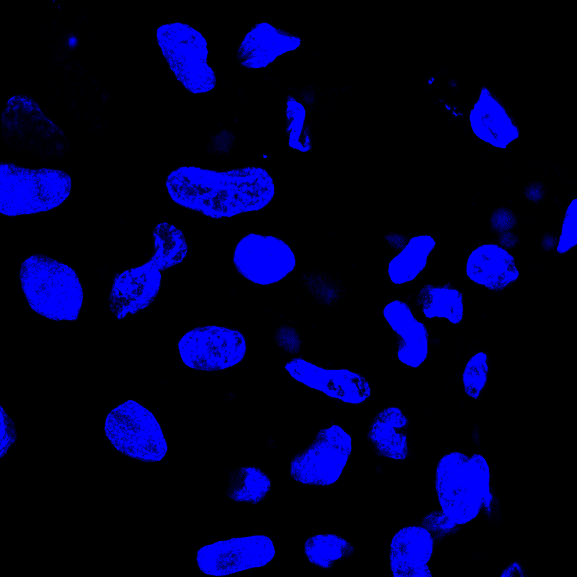


**RGV-27R**

**RGV-91R**

**Hoechst**

**Merge**

5 μm

5 μm

**12 hpi**

**24 hpi**

**RGV**

C

**Fig. S6 Temporal expression and localization of RGV-24R and RGV-91R**. A. Western blot analysis of vHelicase/primase (RGV-24R) and vPCNA (RGV-91R). GSTC cells were infected by RGV at 0.5 MOI and collected at indicated time points. B. Localization of RGV-24R by immunofluorescence in GSTC cells. RGV-24R (green) colocalized with the vSSB (red). Representative overlaps are indicated by white arrows. C. Localization of RGV-91R by immunofluorescence. RGV-91R (green) colocalized with the vSSB (red). Representative overlaps are indicated by white arrows. The visible Hoechst-labeled cytoplasmic viral factories are indicated with yellow arrows.

Rpb3

EdU

DAPI

Merge


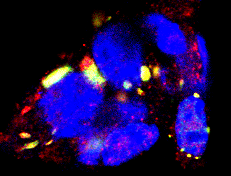

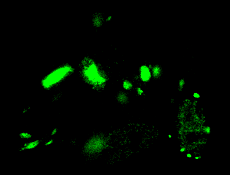

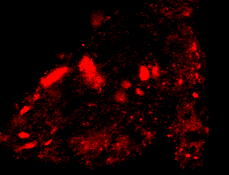

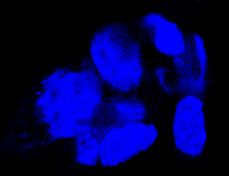


RGV-12h


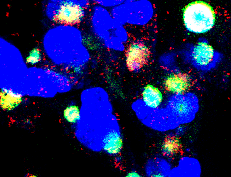

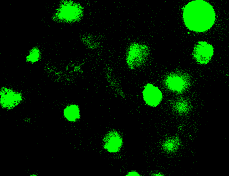

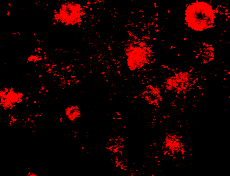

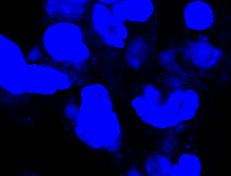


RGV-24h

A

Rpb6

EdU

DAPI

Merge


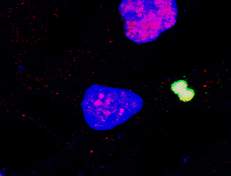

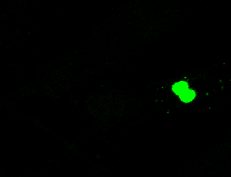

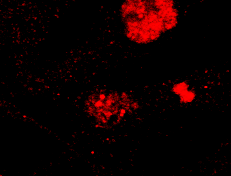

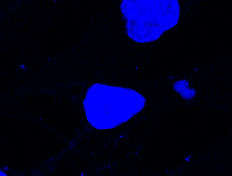


RGV-12h


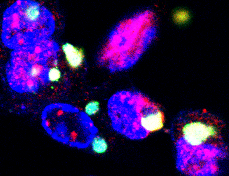

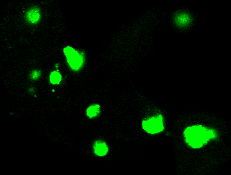

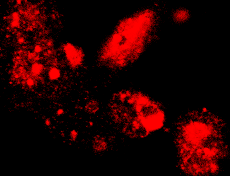

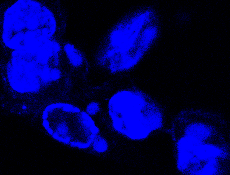


RGV-24h

B

EdU

Rpb11

DAPI

Merge


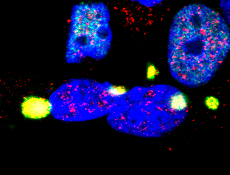

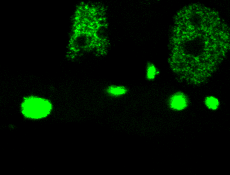

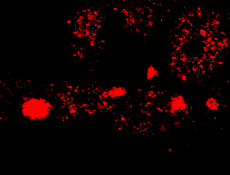

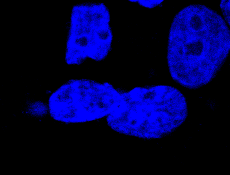


RGV-12h


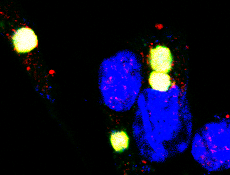

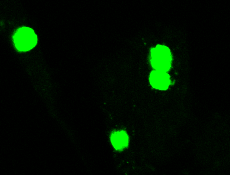

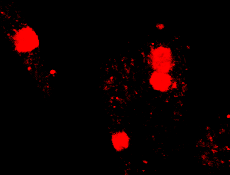

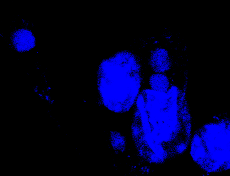


RGV-24h

C

**Fig. S7 Localization of Rpb3, Rpb6, and Rpb11 in RGV infected BHK-21 cells.** The cells were infected with RGV at an MOI of 0.5 and fixed at the indicated time points. Rpb3, Rpb6, and Rpb11 (red) were stained with commercial antibodies. Replicating DNA was stained with EdU (green) as described above. Cell nuclei were stained with DAPI (blue). Bar=5 μM. Rpb3, Rpb6, and Rpb11 were located in viral factories during RGV infection.


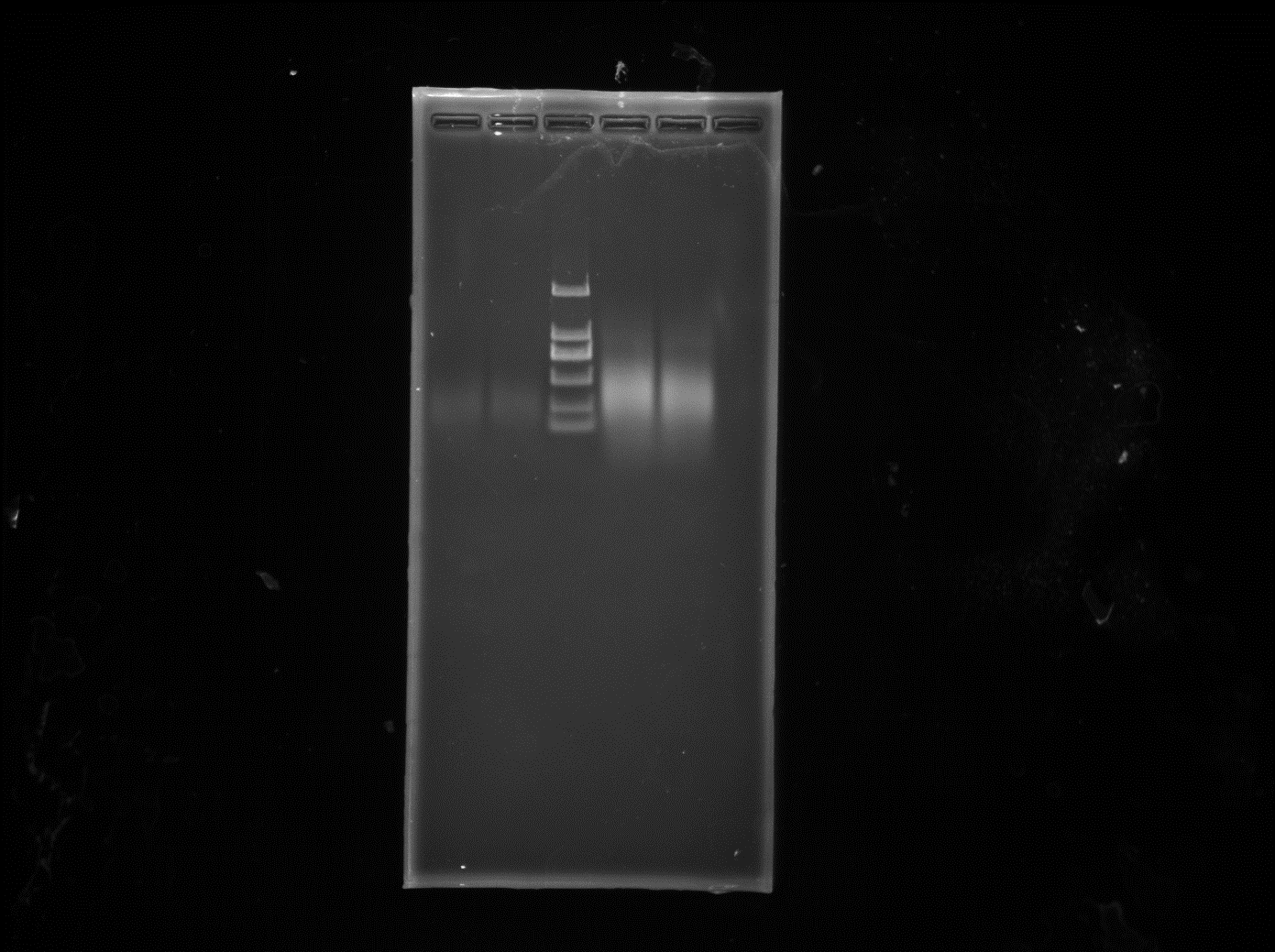


M 1 2

bp

2000

1000

750

500

250

100

**Fig. S8 Gel electrophoresis of the deproteinized DNA.** In the iPOND assay, cross-links were reversed in lysates collected after DNA sonication. The bound proteins were digested, and the DNA fragments were separated on a 1% agarose gel. The main molecular weights of the DNA fragments in the present study are from 250 to 500 bp.
