# Supplementary material for: Replication and transcription machinery for ranaviruses: components, correlation, and functional architecture
Source: Cell Biosci. 2022 Jan 6;12:6. doi: 10.1186/s13578-021-00742-x (PMC8734342; doi:10.1186/s13578-021-00742-x)
Supplement: Supplementary file 2 — Additional file 2: Table S1. Primers and constructs used in the study. [file 13578_2021_742_MOESM2_ESM.docx]

| Primers | Sequences (5'-3')  Table S1. Primers and constructs used in the study | Constructs | Applications |
| --- | --- | --- | --- |
| ICP18-F | ACGACCTGGGAACCTTTGTG |  | qPCR |
| ICP18-R | GTTGTCGTACAGGCATTCGC |  |  |
| MCP-F | CACCTCCATCCCAGTCAGCA | pMD18T-MCP | qPCR |
| MCP-R | AATCCCATCGAGCCGTTCA |  |  |
| 32a-85L-F | CAACCATGGCAATGGATCCCACAAG | pET32a-85L/ pET32a-27R | EMSA, antibody preparation |
| 32a-85L-R | TTTTGAATTCCGTCTTACTGATCC |  |  |
| 32a-23L-F | CAACCATGGATGCTGTGGGAAG | pET32a-23L | Antibody preparation |
| 32a-23L-R | CGCCGAATTCTTCAATACATTAG |  |  |
| C5x-88L-F | TCACATATGATCCCCAAGGCCAGG | pMAL-c5x-88L | Antibody preparation |
| C5x-88L-R | GACCTGGAATTCTCAGTCCTACTCT |  |  |
| HAM-23L-F | AGCCTGATGCTGATGTCTAGAATGCTGTGGGAAGCCGT | pCGN-HAM-23L | Co-IP |
| HAM-23L-R | ACCCTGAAGTTCTCAGGATCCTTAGCCCTCAAAGAGAGT |  |  |
| HAM-85L-F | AGCCTGATGCTGATGTCTAGAATGGATCCCACAAGC | pCGN-HAM-85L |  |
| HAM-85L-R | ACCCTGAAGTTCTCAGGATCCTTACTGATCCGCCTC |  |  |
| HAM-88L-F | AGCCTGATGCTGATGTCTAGAATGGCTAACGCTACC | pCGN-HAM-88L |  |
| HAM-88L-R | ACCCTGAAGTTCTCAGGATCCCTACTCTTGCTGCTCG |  |  |
| HAM-TopIIβ-F | AGCCTGATGCTGATGTCTAGAATGTCCAAAGCCGACG | pCGN-HAM-topoisomerase IIβ |  |
| HAM-TopIIβ-R | ACCCTGAAGTTCTCAGGATCCTTAAGTAAACATATCAAAATC |  |  |
| 47L-3Flag-F | CGATAAGGCCCGGGCGGATCCCATGGATCTCTTTGTG | pcDNA3.1-47L- 3Flag |  |
| 47L-3Flag-R | CGATAAGCTTGATATCGAATTCTTACTTTTTCTTGAACGACAC |  |  |
| 88L-3Flag-F | CGATAAGGCCCGGGCGGATCCCATGGCTAACGCTACC | pcDNA3.1-88L- 3Flag |  |
| 88L-3Flag-R | CGATAAGCTTGATATCGAATTCCTACTCTTGCTGCTCG |  |  |
| 85L-3Flag-F | CGATAAGGCCCGGGCGGATCCCATGGATCCCACAAGC | pcDNA3.1-85L- 3Flag |  |
| 85L-3Flag-R | CGATAAGCTTGATATCGAATTCTTACTGATCCGCCTC |  |  |
| Rpb3-3Flag-F | TGCTGGATATCTGCAGAATTCAGCATGGGAATGCCTTACGCCAATCAG | pcDNA3.1-Rpb3-3Flag |  |
| Rpb3-3Flag-R | ATCCTTGTAGTCACTTAAGCTGTTAATGGTGAGTACGTC |  |  |
| Rpb6-3Flag-F | TGCTGGATATCTGCAGAATTCAGCATGGGAATGTCTGACAACGAGGAC | pcDNA3.1-Rpb6-3Flag |  |
| Rpb6-3Flag-R | ATCCTTGTAGTCACTTAAGCTGTCAGTGATGATCAGCTC |  |  |
| Rpb11-3Flag-F | TGCTGGATATCTGCAGAATTCAGCATGGGAATGAATGCGCCGCCTGCT | pcDNA3.1-Rpb11-3Flag |  |
| Rpb11-3Flag-R | ATCCTTGTAGTCACTTAAGCTTTCAATTCCCTCCTGTTTG |  |  |
| TopIIα-3Flag-F | TGCTGGATATCTGCAGAATTCAAAATGGAGACCGCCGAG | pcDNA3.1-TopIIα-3Flag |  |
| TopIIα-3Flag-R | ATCCTTGTAGTCACTTAAGCTACAGAACATATCCTCG |  |  |
| NlucN-47L-F | GAGGCTCTGGAGGGGAATTCATGGATCTCTTTGTGTACCAGTGG | pcDNA-NlucN-47L | NanoLuc complementation assay |
| NlucN-47L-R | TCATCCTTGTAGTCGGATCCCTTTTTCTTGAACGACACAATCTT |  |  |
| 47L-NlucN-F | CCAAGCTGGCTAGCGAATTCATGGATCTCTTTGTGTACCAGTGG | pcDNA-47L-NlucN |  |
| 47L-NlucN-R | CCACCGCTGCCACCGGATCCCTTTTTCTTGAACGACACAATCTT |  |  |
| NlucN-Rpb3-F | GAGGCTCTGGAGGGGAATTCATGCCTTACGCCAATCAG | pcDNA-NlucN-Rpb3 |  |
| NlucN-Rpb3-R | TCATCCTTGTAGTCGGATCCGTTAATGGTGAGTACGTC |  |  |
| Rpb3-NlucN-F | GGGAGACCCAAGCTGGCTAGCATGGGAATGCCTTACGCCAATCAG | pcDNA-Rpb3-NlucN |  |
| Rpb3-NlucN-R | CCACCGCTGCCACCGGATCCGTTAATGGTGAGTACGTC |  |  |
| NlucN-Rpb6-F | GAGGCTCTGGAGGGGAATTCATGTCTGACAACGAGGAC | pcDNA-NlucN-Rpb6 |  |
| NucN-Rpb6-R | TCATCCTTGTAGTCGGATCCGTCAGTGATGATCAGCTC |  |  |
| Rpb6-NlucN-F | GGGAGACCCAAGCTGGCTAGCATGGGAATGTCTGACAACGAGGAC | pcDNA-Rpb6-NlucN |  |
| Rpb6-NlucN-R | CCACCGCTGCCACCGGATCCGTCAGTGATGATCAGCTC |  |  |
| NlucN-Rpb11-F | GAGGCTCTGGAGGGGAATTCATGAATGCGCCGCCTGCT | pcDNA-NlucN-Rpb11 |  |
| NlucN-Rpb11-R | TCATCCTTGTAGTCGGATCCTTCAATTCCCTCCTGTTTG |  |  |
| Rpb11-NlucN-F | GGGAGACCCAAGCTGGCTAGCATGGGAATGAATGCGCCGCCTGCT | pcDNA-Rpb11-NlucN |  |
| Rpb11-NlucN-R | CCACCGCTGCCACCGGATCCTTCAATTCCCTCCTGTTTG |  |  |
| NlucC-1R-F | GGGGCTCATCGGGGGAATTCATGGCATTCTCGACAGAAG | pcDNA-NlucC-1R | NanoLuc complementation assay, co-IP |
| NlucC-1R-R | CACATCATAGGGGTAGGATCCCAGGGGGGTAAACTTCC |  |  |
| 1R-NlucC-F | GGGAGACCCAAGCTGGCTAGCATGGCATTCTCGACAGAAG | pcDNA-1R-NlucC |  |
| 1R-NlucC-R | GGAGCCGCTGTTTCCGGATCCCAGGGGGGTAAACTTCC |  |  |
| NlucC-3L-F | GGGGCTCATCGGGGGAATTCATGCCATTTTCTGACAG | pcDNA-NlucC-3L |  |
| NlucC-3L-R | CACATCATAGGGGTAGGATCCACAGTCTGGGGTCCAG |  |  |
| 3L-NlucC-F | GGGAGACCCAAGCTGGCTAGCATGGGAATGCCATTTTCTGACAG | pcDNA-3L-NlucC |  |
| 3L-NlucC-R | GGAGCCGCTGTTTCCGGATCCACAGTCTGGGGTCCAG |  |  |
| NlucC-5R-F | GGGGCTCATCGGGGGAATTCATGAACGCAAAATACG | pcDNA-NlucC-5R |  |
| NlucC-5R-R | CACATCATAGGGGTAGGATCCATATCTATATCTGCTAG |  |  |
| 5R-NlucC-F | GGGAGACCCAAGCTGGCTAGCATGGGAATGAACGCAAAATACG | pcDNA-5R-NlucC |  |
| 5R-NlucC-R | GGAGCCGCTGTTTCCGGATCCATATCTATATCTGCTAG |  |  |
| NlucC-6R-F | GGGGCTCATCGGGGGAATTCATGTATATCCCAAGA | pcDNA-NlucC-6R |  |
| NlucC-6R-R | CACATCATAGGGGTAGGATCCAACTTTTGGTCCTTGAG |  |  |
| 6R-NlucC-F | GGGAGACCCAAGCTGGCTAGCATGGGAATGTATATCCCAAGA | pcDNA-6R-NlucC |  |
| 6R-NlucC-R: | GGAGCCGCTGTTTCCGGATCCAACTTTTGGTCCTTGAG |  |  |
| NlucC-9R-F | GGGGCTCATCGGGGGAATTCATGGAAATGTTTGCATC | pcDNA-NlucC-9R |  |
| NlucC-9R-R | CACATCATAGGGGTAGGATCCTCGCCACTCAAAGGATTC |  |  |
| 9R-NlucC-F | GGGAGACCCAAGCTGGCTAGCATGGAAATGTTTGCATC | pcDNA-9R-NlucC |  |
| 9R-NlucC-R | GGAGCCGCTGTTTCCGGATCCTCGCCACTCAAAGGATTC |  |  |
| NlucC-10L-F | GGGGCTCATCGGGGGAATTCATGGACACGTCACCCT | pcDNA-NlucC-10L |  |
| NlucC-10L-R | CACATCATAGGGGTAGGATCCGGCAAACTTGCCCCTC |  |  |
| 10L-NlucC-F | GGGAGACCCAAGCTGGCTAGCATGGACACGTCACCCT | pcDNA-10L-NlucC |  |
| 10L-NlucC-R | GGAGCCGCTGTTTCCGGATCCGGCAAACTTGCCCCTC |  |  |
| NlucC-12L-F | GGGGCTCATCGGGGGAATTCATGGGCATAAAAGGAC | pcDNA-NlucC-12L |  |
| NlucC-12L-R | CACATCATAGGGGTAGGATCCCTTGCGCTTGCACTTC |  |  |
| 12L-NlucC-F | GGGAGACCCAAGCTGGCTAGCATGGGCATAAAAGGAC | pcDNA-12L-NlucC |  |
| 12L-NlucC-R | GGAGCCGCTGTTTCCGGATCCCTTGCGCTTGCACTTC |  |  |
| NlucC-19L-F | GGGGCTCATCGGGGGAATTCATGCACGGTTGCAATTG | pcDNA-NlucC-19L |  |
| NlucC-19L-R | CACATCATAGGGGTAGGATCCGTTAAAAGTGCTCGTAT |  |  |
| 19L-NlucC-F | GGGAGACCCAAGCTGGCTAGCATGGGAATGCACGGTTGCAATTG | pcDNA-19L-NlucC |  |
| 19L-NlucC-R | GGAGCCGCTGTTTCCGGATCCGTTAAAAGTGCTCGTAT |  |  |
| NlucC-20R-F | GGGGCTCATCGGGGGAATTCATGTACCTAAACGCG | pcDNA-NlucC-20R |  |
| NlucC-20R-R | CACATCATAGGGGTAGGATCCTTCCATATCGTTGTCG |  |  |
| 20R-NlucC-F | GGGAGACCCAAGCTGGCTAGCATGGGAATGTACCTAAACGCG | pcDNA-20R-NlucC |  |
| 20R-NlucC-R | GGAGCCGCTGTTTCCGGATCCTTCCATATCGTTGTCG |  |  |
| NlucC-23L-F | GGGGCTCATCGGGGGAATTCATGCTGTGGGAAGC | pcDNA-NlucC-23L |  |
| NlucC-23L-R | CACATCATAGGGGTAGGATCCGCCCTCAAAGAGAG |  |  |
| 23L-NlucC-F | GGGAGACCCAAGCTGGCTAGCATGGGAATGCTGTGGGAAGC | pcDNA-23L-NlucC |  |
| 23L-NlucC-R | GGAGCCGCTGTTTCCGGATCCGCCCTCAAAGAGAG |  |  |
| NlucC-24L-F | GGGGCTCATCGGGGGAATTCATGAGGATACTGGATC | pcDNA-NlucC-24L |  |
| NlucC-24L-R | CACATCATAGGGGTAGGATCCGCACAGGTCCAACAGC |  |  |
| 24L-NlucC-F | GGGAGACCCAAGCTGGCTAGCATGGGAATGAGGATACTGGATC | pcDNA-24L-NlucC |  |
| 24L-NlucC-R | GGAGCCGCTGTTTCCGGATCCGCACAGGTCCAACAGC |  |  |
| NlucC-26L-F | GGGGCTCATCGGGGGAATTCATGAGCATGATCCAAG | pcDNA-NlucC-26L |  |
| NlucC-26L-R | CACATCATAGGGGTAGGATCCTACACCCTCCACGAC |  |  |
| 26L-NlucC-F | GGGAGACCCAAGCTGGCTAGCATGGGAATGAGCATGATCCAAG | pcDNA-26L-NlucC |  |
| 26L-NlucC-R | GGAGCCGCTGTTTCCGGATCCTACACCCTCCACGAC |  |  |
| NlucC-27L-F | GGGGCTCATCGGGGGAATTCATGTCTTTTCAGAGAGATTACG | pcDNA-NlucC-27L |  |
| NlucC-27L-R | CACATCATAGGGGTAGGATCCCCTGGTCCACCTCTTGC |  |  |
| 27L-NlucC-F | GGGAGACCCAAGCTGGCTAGCATGGGAATGTCTTTTCAGAGAGATTACG | pcDNA-27L-NlucC |  |
| 27L-NlucC-R | GGAGCCGCTGTTTCCGGATCCCCTGGTCCACCTCTTGC |  |  |
| NlucC-28R-F | GGGGCTCATCGGGGGAATTCATGGAAGGTTGGTTG | pcDNA-NlucC-28R |  |
| NlucC-28R-R | CACATCATAGGGGTAGGATCCGACTCCCTTGGCATG |  |  |
| 28R-NlucC-F | GGGAGACCCAAGCTGGCTAGCATGGAAGGTTGGTTG | pcDNA-28R-NlucC |  |
| 28R-NlucC-R | GGAGCCGCTGTTTCCGGATCCGACTCCCTTGGCATG |  |  |
| NlucC-29L-F | GGGGCTCATCGGGGGAATTCATGTTGGACATGAGATC | pcDNA-NlucC-29L |  |
| NlucC-29L-R | CACATCATAGGGGTAGGATCCCACCTCAAACACGTCG |  |  |
| 29L-NlucC-F | GGGAGACCCAAGCTGGCTAGCATGGGAATGTTGGACATGAGATC | pcDNA-29L-NlucC |  |
| 29L-NlucC-R | GGAGCCGCTGTTTCCGGATCCCACCTCAAACACGTCG |  |  |
| NlucC-31R-F | GGGGCTCATCGGGGGAATTCATGGTCATGTGCTGGTGG | pcDNA-NlucC-31R |  |
| NlucC-31R-R | CACATCATAGGGGTAGGATCCGACGGGCCACACGGCC |  |  |
| 31R-NlucC-F | GGGAGACCCAAGCTGGCTAGCATGGTCATGTGCTGGTGG | pcDNA-31R-NlucC |  |
| 31R-NlucC-R | GGAGCCGCTGTTTCCGGATCCGACGGGCCACACGGCC |  |  |
| NlucC-35R-F | GGGGCTCATCGGGGGAATTCATGCTTATCTCATTGTCTGAG | pcDNA-NlucC-35R |  |
| NlucC-35R-R | CACATCATAGGGGTAGGATCCGATAATGTTAAACTTTGTCAGG |  |  |
| 35R-NlucC-F | GGGAGACCCAAGCTGGCTAGCATGGGAATGCTTATCTCATTGTCTGAG | pcDNA-35R-NlucC |  |
| 35R-NlucC-R | GGAGCCGCTGTTTCCGGATCCGATAATGTTAAACTTTGTCAGG |  |  |
| NlucC-38L-F | GGGGCTCATCGGGGGAATTCATGGCTTCTCACTAC | pcDNA-NlucC-38L |  |
| NlucC-38L-R | CACATCATAGGGGTAGGATCCCCTGAATCGGCC |  |  |
| 38L-NlucC-F | GGGAGACCCAAGCTGGCTAGCATGGCTTCTCACTAC | pcDNA-38L-NlucC |  |
| 38L-NlucC-R | GGAGCCGCTGTTTCCGGATCCCCTGAATCGGCC |  |  |
| NlucC-45L-F | GGGGCTCATCGGGGGAATTCATGTTTGCACTTGAAAC | pcDNA-NlucC-45L |  |
| NlucC-45L-R | CACATCATAGGGGTAGGATCCAGCCTGTTCGGGGTT |  |  |
| 45L-NlucC-F | GGGAGACCCAAGCTGGCTAGCATGGGAATGTTTGCACTTGAAAC | pcDNA-45L-NlucC |  |
| 45L-NlucC-R | GGAGCCGCTGTTTCCGGATCCAGCCTGTTCGGGGTT |  |  |
| NlucC-46R-F | GGGGCTCATCGGGGGAATTCATGTCCAGGGGCATG | pcDNA-NlucC-46R |  |
| NlucC-46R-R | CACATCATAGGGGTAGGATCCCTTGAAGGCTATGGAAATC |  |  |
| 46R-NlucC-F | GGGAGACCCAAGCTGGCTAGCATGGGAATGTCCAGGGGCATG | pcDNA-46R-NlucC |  |
| 46R-NlucC-R | GGAGCCGCTGTTTCCGGATCCCTTGAAGGCTATGGAAATC |  |  |
| NlucC-50L-F | GGGGCTCATCGGGGGAATTCATGAGGATCTTTGGAG | pcDNA-NlucC-50L |  |
| NlucC-50L-R | CACATCATAGGGGTAGGATCCCTTTCTCTTGTCCATCC |  |  |
| 50L-NlucC-F | GGGAGACCCAAGCTGGCTAGCATGGGAATGAGGATCTTTGGAG | pcDNA-50L-NlucC |  |
| 50L-NlucC-R | GGAGCCGCTGTTTCCGGATCCCTTTCTCTTGTCCATCC |  |  |
| NlucC-51L-F | GGGGCTCATCGGGGGAATTCATGGCAATGGTTTCC | pcDNA-NlucC-51L |  |
| NlucC-51L-R | CACATCATAGGGGTAGGATCCCAGGCTCTTTAGGAT |  |  |
| 51L-NlucC-F | GGGAGACCCAAGCTGGCTAGCATGGCAATGGTTTCC | pcDNA-51L-NlucC |  |
| 51L-NlucC-R | GGAGCCGCTGTTTCCGGATCCCAGGCTCTTTAGGAT |  |  |
| NlucC-61L-F | GGGGCTCATCGGGGGAATTCATGACTGTCCAGATTAC | pcDNA-NlucC-61L |  |
| NlucC-61L-R | CACATCATAGGGGTAGGATCCAGATTTCATCGCTAGC |  |  |
| 61L-NlucC-F | GGGAGACCCAAGCTGGCTAGCATGGGAATGACTGTCCAGATTAC | pcDNA-61L-NlucC |  |
| 61L-NlucC-R | GGAGCCGCTGTTTCCGGATCCAGATTTCATCGCTAGC |  |  |
| NlucC-62R-F | GGGGCTCATCGGGGGAATTCATGCAAGTCTACTCTC | pcDNA-NlucC-62R |  |
| NlucC-62R-R | CACATCATAGGGGTAGGATCCACACAGATAATCTTCAG |  |  |
| 62R-NlucC-F | GGGAGACCCAAGCTGGCTAGCATGGGAATGCAAGTCTACTCTC | pcDNA-62R-NlucC |  |
| 62R-NlucC-R | GGAGCCGCTGTTTCCGGATCCACACAGATAATCTTCAG |  |  |
| NlucC-64R-F | GGGGCTCATCGGGGGAATTCATGCATACCATTTCAG | pcDNA-NlucC-64R |  |
| NlucC-64R-R | CACATCATAGGGGTAGGATCCAAGGTCCAGTTCG |  |  |
| 64R-NlucC-F | GGGAGACCCAAGCTGGCTAGCATGGGAATGCATACCATTTCAG | pcDNA-64R-NlucC |  |
| 64R-NlucC-R | GGAGCCGCTGTTTCCGGATCCAAGGTCCAGTTCG |  |  |
| NlucC-66R-F | GGGGCTCATCGGGGGAATTCATGGACGATGTCGAGT | pcDNA-NlucC-66R |  |
| NlucC-66R-R | CACATCATAGGGGTAGGATCCTTTTAATGCTAACCACAGC |  |  |
| 66R-NlucC-F | GGGAGACCCAAGCTGGCTAGCATGGACGATGTCGAGT | pcDNA-66R-NlucC |  |
| 66R-NlucC-R | GGAGCCGCTGTTTCCGGATCCTTTTAATGCTAACCACAGC |  |  |
| NlucC-68L-F | GGGGCTCATCGGGGGAATTCATGAGAGTCGTGGTAAAC | pcDNA-NlucC-68L |  |
| NlucC-68L-R | CACATCATAGGGGTAGGATCCCATCAGAAGAGACAC |  |  |
| 68L-NlucC-F | GGGAGACCCAAGCTGGCTAGCATGGGAATGAGAGTCGTGGTAAAC | pcDNA-68L-NlucC |  |
| 68L-NlucC-R | GGAGCCGCTGTTTCCGGATCCCATCAGAAGAGACAC |  |  |
| NlucC-79L-F | GGGGCTCATCGGGGGAATTCATGGTTACTGTTACTG | pcDNA-NlucC-79L |  |
| NlucC-79L-R | CACATCATAGGGGTAGGATCCGTATCCCAGAGCC |  |  |
| 79L-NlucC-F | GGGAGACCCAAGCTGGCTAGCATGGTTACTGTTACTG | pcDNA-79L-NlucC |  |
| 79L-NlucC-R | GGAGCCGCTGTTTCCGGATCCGTATCCCAGAGCC |  |  |
| NlucC-82L-F | GGGGCTCATCGGGGGAATTCATGTGCCGCTTTGCCTCA | pcDNA-NlucC-82L |  |
| NlucC-82L-R | CACATCATAGGGGTAGGATCCGCACACTATGTAAACGTC |  |  |
| 82L-NlucC-F | GGGAGACCCAAGCTGGCTAGCATGGGAATGTGCCGCTTTGCCTCA | pcDNA-82L-NlucC |  |
| 82L-NlucC-R | GGAGCCGCTGTTTCCGGATCCGCACACTATGTAAACGTC |  |  |
| NlucC-83L-F | GGGGCTCATCGGGGGAATTCATGGCCAATTTTCTAC | pcDNA-NlucC-83L |  |
| NlucC-83L-R | CACATCATAGGGGTAGGATCCGCCCCGACCGTCCT |  |  |
| 83L-NlucC-F | GGGAGACCCAAGCTGGCTAGCATGGCCAATTTTCTAC | pcDNA-83L-NlucC |  |
| 83L-NlucC-R | GGAGCCGCTGTTTCCGGATCCGCCCCGACCGTCCT |  |  |
| NlucC-85L-F | GGGGCTCATCGGGGGAATTCATGGATCCCACAAGC | pcDNA-NlucC-85L |  |
| NlucC-85L-R | CACATCATAGGGGTAGGATCCCTGATCCGCCTCCTC |  |  |
| 85L-NlucC-F | GGGAGACCCAAGCTGGCTAGCATGGATCCCACAAGC | pcDNA-85L-NlucC |  |
| 85L-NlucC-R | GGAGCCGCTGTTTCCGGATCCCTGATCCGCCTCCTC |  |  |
| NlucC-86L-F | GGGGCTCATCGGGGGAATTCATGTGGCAGTACTTAC | pcDNA-NlucC-86L |  |
| NlucC-86L-R | CACATCATAGGGGTAGGATCCCTTGTTCAGAGAGAC |  |  |
| 86L-NlucC-F | GGGAGACCCAAGCTGGCTAGCATGGGAATGTGGCAGTACTTAC | pcDNA-86L-NlucC |  |
| 86L-NlucC-R | GGAGCCGCTGTTTCCGGATCCCTTGTTCAGAGAGAC |  |  |
| NlucC-88L-F | GGGGCTCATCGGGGGAATTCATGGCTAACGCTACC | pcDNA-NlucC-88L |  |
| NlucC-88L-R | CACATCATAGGGGTAGGATCCCTCTTGCTGCTCGG |  |  |
| 88L-NlucC-F | GGGAGACCCAAGCTGGCTAGCATGGCTAACGCTACC | pcDNA-88L-NlucC |  |
| 88L-NlucC-R | GGAGCCGCTGTTTCCGGATCCCTCTTGCTGCTCGG |  |  |
| NlucC-89R-F | GGGGCTCATCGGGGGAATTCATGGAAACCATAGTG | pcDNA-NlucC-89R |  |
| NlucC-89R-R | CACATCATAGGGGTAGGATCCCGACGAGGACCCAAA |  |  |
| 89R-NlucC-F | GGGAGACCCAAGCTGGCTAGCATGGAAACCATAGTG | pcDNA-89R-NlucC |  |
| 89R-NlucC-R | GGAGCCGCTGTTTCCGGATCCCGACGAGGACCCAAA |  |  |
| NlucC-91L-F | GGGGCTCATCGGGGGAATTCATGACAAACAGGGTC | pcDNA-NlucC-91L |  |
| NlucC-91L-R | CACATCATAGGGGTAGGATCCCATCGTGAAGCTCTC |  |  |
| 91L-NlucC-F | GGGAGACCCAAGCTGGCTAGCATGGGAATGACAAACAGGGTC | pcDNA-91L-NlucC |  |
| 91L-NlucC-R | GGAGCCGCTGTTTCCGGATCCCATCGTGAAGCTCTC |  |  |
| NlucC-94R-F | GGGGCTCATCGGGGGAATTCATGAGAAACCTTTTGA | pcDNA-NlucC-94R |  |
| NlucC-94R-R | CACATCATAGGGGTAGGATCCACCCGCAAAATACTTC |  |  |
| 94R-NlucC-F | GGGAGACCCAAGCTGGCTAGCATGGGAATGAGAAACCTTTTGA | pcDNA-94R-NlucC |  |
| 94R-NlucC-R | GGAGCCGCTGTTTCCGGATCCACCCGCAAAATACTTC |  |  |
| NlucC-96L-F | GGGGCTCATCGGGGGAATTCATGGAACAAGTACC | pcDNA-NlucC-96L |  |
| NlucC-96L-R | CACATCATAGGGGTAGGATCCATCGTCCAAGTCCGA |  |  |
| 96L-NlucC-F | GGGAGACCCAAGCTGGCTAGCATGGAACAAGTACC | pcDNA-96L-NlucC |  |
| 96L-NlucC-R | GGAGCCGCTGTTTCCGGATCCATCGTCCAAGTCCGA |  |  |
| NlucC-97L-F | GGGGCTCATCGGGGGAATTCATGGAAACGTTGG | pcDNA-NlucC-97L |  |
| NlucC-97L-R | CACATCATAGGGGTAGGATCCCTCGTCGTCGTCTTC |  |  |
| 97L-NlucC-F | GGGAGACCCAAGCTGGCTAGCATGGAAACGTTGG | pcDNA-97L-NlucC |  |
| 97L-NlucC-R | GGAGCCGCTGTTTCCGGATCCCTCGTCGTCGTCTTC |  |  |
| NlucC-98R-F | GGGGCTCATCGGGGGAATTCATGTGCTCCAAACTCG | pcDNA-NlucC-98R |  |
| NlucC-98R-R | CACATCATAGGGGTAGGATCCGAAACCCATGGTCT |  |  |
| 98R-NlucC-F | GGGAGACCCAAGCTGGCTAGCATGGGAATGTGCTCCAAACTCG | pcDNA-98R-NlucC |  |
| 98R-NlucC-R | GGAGCCGCTGTTTCCGGATCCGAAACCCATGGTCT |  |  |

The restriction enzyme sites were labeled with underline.
