# Supplementary material for: Replication and transcription machinery for ranaviruses: components, correlation, and functional architecture
Source: Cell Biosci. 2022 Jan 6;12:6. doi: 10.1186/s13578-021-00742-x (PMC8734342; doi:10.1186/s13578-021-00742-x)
Supplement: Supplementary file 3 — Additional file 3: Table S2. Sequences of the siRNAs used in the study. [file 13578_2021_742_MOESM3_ESM.docx]

Table S2 Sequences of the siRNAs used in the present study

| siRNAs | Positions in the gene | Sense sequence (5’-3’) | Anti-sense sequence (5’-3’) |
| --- | --- | --- | --- |
| siRpb3-1 | *Rpb3*-123 | GGUGCCCAUAAUAGCCAUUTT | AAUGGCUAUUAUGGGCACCTT |
| siRpb3-2 | *Rpb3*-429 | GGAGCAGGAUGACAUCCUUTT | AAGGAUGUCAUCCUGCUCCTT |
| siRpb3-3 | *Rpb3*-609 | GCCAAAGAGUGAGUACUCUTT | AGAGUACUCACUCUUUGGCTT |
| siRpb6-1 | *Rpb6*-48 | GGAAGAGGACGAAGGACUATT | UAGUCCUUCGUCCUCUUCCTT |
| siRpb6-2 | *Rpb6*-152 | GCAUCACUACUCCUUACAUTT | AUGUAAGGAGUAGUGAUGCTT |
| siRpb6-3 | *Rpb6*-300 | GAAGAUCCCAAUCAUCAUUTT | AAUGAUGAUUGGGAUCUUCTT |
| siRpb11-1 | *Rpb11*-69 | GGACACAAAGGUGCCCAAUTT | AUUGGGCACCUUUGUGUCCTT |
| siRpb11-2 | *Rpb11*-147 | GCUGUUGAAGGACCCACAGTT | CUGUGGGUCCUUCAACAGCTT |
| siRpb11-3 | *Rpb11*-321 | GGCCAUCAAGGACAAGCAATT | UUGCUUGUCCUUGAUGGCCTT |
| NC |  | UUCUCCGAACGUGUCACGUTT | ACGUGACACGUUCGGAGAATT |
